# *Cy-1*, a major QTL for tomato leaf curl New Delhi virus resistance, harbors a gene encoding a DFDGD-Class RNA-dependent RNA polymerase in cucumber (*Cucumis sativus*)

**DOI:** 10.1186/s12870-024-05591-7

**Published:** 2024-10-02

**Authors:** Sota Koeda, Chihiro Yamamoto, Hiroto Yamamoto, Kohei Fujishiro, Ryoma Mori, Momoka Okamoto, Atsushi J. Nagano, Takaaki Mashiko

**Affiliations:** 1 Graduate School of Agriculture, Kindai University, Nara, Nara 631-8505 Japan; 2 Faculty of Agriculture, Kindai University, Nara, Nara 631-8505 Japan; 3 Faculty of Agriculture, Ryukoku University, Otsu, Shiga 520-2914 Japan; 4 Institute for Advanced Biosciences, Keio University, Tsuruoka, Yamagata 997-0017 Japan; 5 Takii & Co. Ltd, Konan, Shiga 520-3231 Japan

**Keywords:** Begomovirus, Cucurbit, Geminivirus, Genetic mapping, Marker-assisted breeding, Resistance gene, RDR, ToLCNDV, Virus-induced gene silencing (VIGS)

## Abstract

**Background:**

Tomato leaf curl New Delhi virus (ToLCNDV) (family *Geminiviridae*, genus *Begomovirus*) is a significant threat to cucumber (*Cucumis sativus*) production in many regions. Previous studies have reported the genetic mapping of loci related to ToLCNDV resistance, but no resistance genes have been identified.

**Results:**

We conducted map-based cloning of the ToLCNDV resistance gene in cucumber accession No.44. Agroinfiltration and graft-inoculation analyses confirmed the resistance of No.44 to ToLCNDV isolates from the Mediterranean and Asian countries. Initial mapping involving two rounds of phenotyping with two independent F_2_ populations generated by crossing the begomovirus-susceptible cultivar SHF and No.44 consistently detected major quantitative trait loci (QTLs) on chromosomes 1 and 2 that confer resistance to ToLCNDV. Fine-mapping of *Cy-1*, the dominant QTL on chromosome 1, using F_3_ populations narrowed the candidate region to a 209-kb genomic segment harboring 24 predicted genes. Among these genes, *DFDGD-class RNA-dependent RNA polymerase* (*CsRDR3*), an ortholog of *Ty-1*/*Ty-3* of tomato and *Pepy-2* of capsicum, was found to be a strong candidate conferring ToLCNDV resistance. The CsRDR3 sequence of No.44 contained multiple amino acid substitutions; the promoter region of *CsRDR3* in No.44 had a large deletion; and the *CsRDR3* transcript levels were greater in No.44 than in SHF. Virus-induced gene silencing (VIGS) of *CsRDR3* using two chromosome segment substitution lines harboring chromosome 1 segments derived from No.44 compromised resistance to ToLCNDV.

**Conclusions:**

Forward and reverse genetic approaches identified *CsRDR3,* which encodes a DFDGD-class RNA-dependent RNA polymerase, as the gene responsible for ToLCNDV resistance at the major QTL *Cy-1* on chromosome 1 in cucumber. Marker-assisted breeding of ToLCNDV resistance in cucumber will be expedited by using No.44 and the DNA markers developed in this study.

**Supplementary Information:**

The online version contains supplementary material available at 10.1186/s12870-024-05591-7.

## Background

Cucumber (*Cucumis sativus*) cultivated worldwide is an economically important vegetable crop, and its fruit is consumed fresh or pickled [1]. Cucumber is understood to have originated in India and was domesticated in Asia approximately 3,000 years ago [2]. The global production of cucumber was 93.5 million tonnes in 2021, and the largest cucumber producer is China followed by Turkey, Russia, Ukraine, and Mexico [3].

The genus *Begomovirus* (family *Geminiviridae*) contains 445 virus species [4], each of which carries a circular, monopartite or bipartite, single-stranded DNA genome [5]. Most begomoviruses have bipartite genomes organized of two circular DNA components (DNAs A and B), each approximately 2,800 nucleotides (nt) in length [6]. A bipartite begomovirus tomato leaf curl New Delhi virus (ToLCNDV) that infects Solanaceae crops (tomato [*Solanum lycopersicum*], pepper [*Capsicum* spp.], eggplant [*Solanum melongena*], potato [*Solanum tuberosum*]) and cucurbit crops (cucumber, melon [*Cucumis melo*], zucchini squash [*Cucurbita pepo*], luffa [*Luffa* spp.]) [7, 8], is the second most important begomovirus after the monopartite tomato yellow leaf curl virus (TYLCV) and has a high economic impact on horticultural production. ToLCNDV was first detected in India in 1995, and its isolation has subsequently been reported across the Indian subcontinent (India, Pakistan, and Bangladesh) and Southeast Asia (Thailand, Laos, Indonesia, and Taiwan) [9–15]. ToLCNDV mainly threatens the production of cucurbit crops such as cucumber, melon, squash (*Cucurbita* spp.), bottle gourd (*Lagenaria siceraria*), *Luffa* spp., and *Sechium edule* in Asian countries [9, 11, 16–18]. Based on reports from Iran and Turkey in the Middle East and from Spain, Portugal, Italy, Greece, Morocco, Tunisia, and Algeria in the Mediterranean Basin, from 2012 onward, ToLCNDV has spread westward, affecting cucurbit crops such as zucchini squash, melon, and cucumber [19–28].

The insect vector, whitefly *Bemisia tabaci* (Hemiptera: Aleyrodidae), has driven the remarkable emergence of begomoviruses [29]. In tropical and subtropical regions, the distribution and polyphagous feeding habits of *B. tabaci* critically affect the prevalence and economic importance of begomoviral diseases. Generally, insecticides that target *B. tabaci* populations are used to control begomoviruses caused diseases; however, insecticide resistance in *B. tabaci* has emerged by intensive and unregulated use of insecticides [30, 31]. An integrated pest management approach which uses begomovirus-resistant cultivars can be an effective alternative to control begomoviruses. Several begomovirus resistance genes have been cloned from Solanaceae crops using elaborate forward and reverse genetic methods [32–36]. Among the identified resistance genes, *Ty-1*/*Ty-3* in tomato and *Pepy-2* in capsicum encode DFDGD-class RNA-dependent RNA polymerases (RDRs) and are dominant resistance genes to multiple begomoviruses [33, 35]. Furthermore, it was demonstrated that the replication of begomoviral DNA is restricted by RDR via a transcriptional gene silencing pathway [37]. Breeding for begomovirus-resistant tomato plants has advanced, and *Ty-1* has been introgressed into most of the TYLCV-resistant F_1_ hybrid cultivars bred worldwide [38, 39]. Several studies have reported ToLCNDV resistance in cucurbits, such as melon, *C. moschata*, and *L. cylindrica* accessions [40–43]. In cucumber, ToLCNDV-resistant materials and the detection of quantitative trait loci (QTLs) on chromosomes 1 and 2 have been described in international patents from Vilmorin & Cie, Rijk Zwaan, and Nunhems [44–46] More recently, additional ToLCNDV-resistant cucumber accessions were reported and a QTL for resistance was mapped on chromosome 2 [47]. However, no resistance genes have been identified.

In our preliminary study, a total of 575 cucumber accessions were screened for ToLCNDV resistance by agroinfiltration of a Spanish isolate of ToLCNDV (ToLCNDV-ES), and No.44 was selected as one of the resistance sources. In this study, ToLCNDV resistance of a cucumber accession No.44 were evaluated. Reliable QTLs were detected on chromosomes 1 and 2 by two rounds of phenotyping with two independent F_2_ populations generated by crossing the begomovirus-susceptible cultivar SHF and No.44. Further fine-mapping and functional analysis of a candidate gene on chromosome 1 identified *CsRDR3* as the gene responsible for ToLCNDV resistance at the QTL.

## Results

### No.44 is resistant to ToLCNDV isolates from the Mediterranean Basin and Southeast Asia

**Fig. 1:**
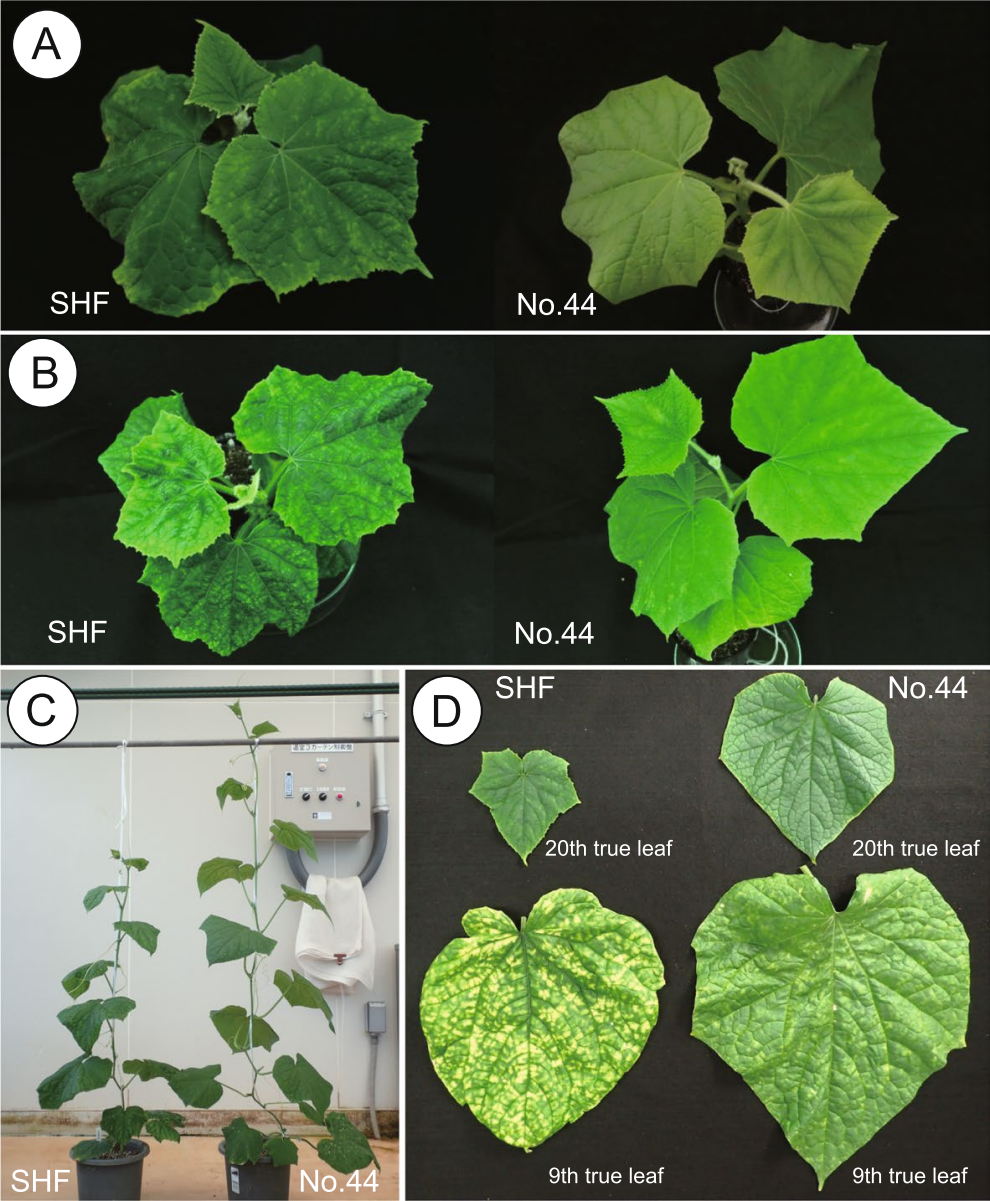
No.44 (resistant) and ‘Sagami Hanjiro Fushinari’ (SHF; susceptible) plants infected with ToLCNDV. **A** ToLCNDV-ES-agroinfiltrated SHF and No.44 plants at 30 days post inoculation (dpi). **B** ToLCNDV-IDN-agroinfiltrated SHF and No.44 plants at 30 dpi. **C** ToLCNDV-ES-infected SHF and No.44 plants at 30 days after graft inoculation. **D** Ninth and 20th leaves of SHF and No.44 plants at 60 days after graft inoculation

**Fig. 2:**
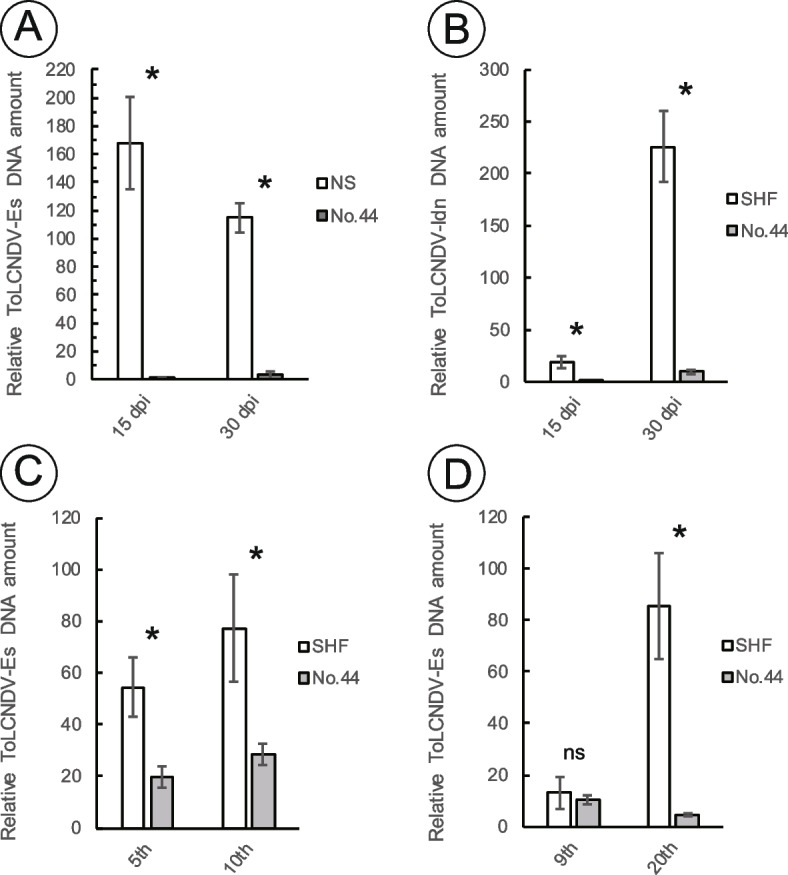
Quantification of ToLCNDV viral DNA in the leaves of cucumber No.44 and susceptible cultivars. Relative amount of viral DNA accumulated in **A** ToLCNDV-ES-agroinfiltrated NS and No.44 plants, **B** ToLCNDV-IDN-agroinfiltrated SHF and No.44 plants, **C** ToLCNDV-ES-infected SHF and No.44 plants after 30 days from graft inoculation, and **D** plants after 60 days from graft inoculation. Only ToLCNDV-infected plants were used for this analysis. NS and SHF indicate the begomovirus-susceptible cultivars ‘Natsu Suzumi’ and ‘Sagami Hanjiro Fushinari’. Young upper leaves of ToLCNDV-infected plants were collected at 15 and 30 days after agroinfiltration and 30 and 60 days after grafting for analysis of viral DNA accumulation by qPCR. Normalized relative viral DNA values were calculated using the 25S rRNA gene. The data are presented as the mean ± standard error (SE). Significant differences between means (Student’s *t* test; *p* < 0.05) are indicated with asterisks

The resistance of cucumber accession No.44 to two ToLCNDV isolates was evaluated by agroinfiltration. In the first experiment, agroinfiltration of ToLCNDV-ES resulted in 100% infection in the susceptible cultivar ‘Sagami Hanjiro Fushinari’ (SHF), and the plants consistently exhibited severe symptoms, with an average disease severity index (DSI) score of 4 (Fig. 1A, Table 1). Moreover, only 26% of the inoculated No.44 plants were virus-infected, and the infected plants showed no symptoms, with an average DSI score of 0 at 30 days post inoculation (dpi). F_1_ showed moderate resistance to ToLCNDV-ES, with an average DSI score of 2.8 at 30 dpi (Table 1). In the second experiment, ToLCNDV-ES was agroinfiltrated into No.44 plants and another susceptible cultivar ‘Natsu Suzumi’ (NS) (Table 1). Again, No.44 was resistant to ToLCNDV-ES, while NS was susceptible to virus infection. Moreover, the accumulation of ToLCNDV-ES viral DNA was significantly lower in No.44 plants than in NS plants (Fig. 2A). In the third experiment, agroinfiltration of ToLCNDV-IDN [15] with higher pathogenicity induced severe symptoms in susceptible SHF plants (Fig. 1B, Table 1). Meanwhile, No.44 was resistant to ToLCNDV-IDN with an average DSI score of 1.4 at 30 dpi. Moreover, the accumulation of ToLCNDV-IDN viral DNA was significantly lower in No.44 plants than in SHF plants (Fig. 2B). From these results, we concluded that No.44 is resistant to ToLCNDV isolates from multiple cucumber-producing regions, including the Mediterranean Basin and Southeast Asia.

**Table 1:** Evaluation of tomato leaf curl New Delhi virus (ToLCNDV) resistance in No.44 and F_1_ population obtained by crossing with SHF

Inoculated virus	Cucumber accession^w^	Number of plants			Disease severity index (DSI) score^Z^	
		Inoculated	Infected^x^	(%)^y^	15 dpi	30 dpi
Exp.1						
ToLCNDV-Es	SHF	10	10	100	4^a^	4^a^
	44	23	6	26	0^c^	0^c^
	SHF×44 F_1_	31	31	100	1.8^b^	2.8^b^
Exp.2						
ToLCNDV-Es	NS	10	10	100	4^a^	4^a^
	44	15	4	27	0^b^	0.3^b^
Exp.3						
ToLCNDV-Idn	SHF	24	24	100	3.2^a^	4^a^
	44	30	14	47	0^b^	1.4^b^

^w^NS = Natsu Suzumi and SHF = Sagami Hanjiro Fushinari (begomovirus susceptible cultivars)

^x^Virus infection was detected by PCR

^y^(Number of plants with infected / number of plants inoculated) × 100

^z^The DSI ranged from a score of 0 to 4 as follows: 0, no symptoms; 1, a few yellow spots on the leaf; 2, yellow spots around the whole leaf; 3, yellowing of the leaf with mild curling; 4, heavy yellowing of the leaf with curling. Different letters indicate significant differences between means (Mann-Whitney U test or Bonferroni-Dunn test, *p* < 0.05)

ToLCNDV-ES was transmitted from a symptomatic NS scion to healthy No.44 or SHF rootstock and evaluated for resistance at 30 and 60 days after grafting. ToLCNDV-ES infection of the NS scion was confirmed by PCR before grafting. At 30 days after grafting, No.44 plants exhibited vigorous growth compared with the susceptible SHF plants (Fig. 1C). The average DSI values of the fifth and 10th true leaves of No.44 plants were significantly lower than those of their counterparts in SHF plants (Table 2). Moreover, at 60 days after grafting, intense yellowing was observed on the ninth true leaf of SHF plants, but only a few spots with mild yellowing were observed on the leaves of No.44, and the average DSI was significantly lower for No.44 than for SHF (Fig. 1D, Table 2). On the 20th true leaf, symptoms were relatively mild in both No.44 and SHF, but the average DSI was significantly lower in No.44 than in SHF (Table 2). Quantification of ToLCNDV-ES DNA in leaves revealed that significantly less ToLCNDV-ES DNA accumulated in the fifth and 10th true leaves of No.44 than in those of SHF at 30 days after grafting (Fig. 2C). At 60 days after grafting, there was no significant difference in the amount of viral DNA in the ninth true leaf between No.44 and SHF plants, but there was significantly less viral DNA in the 20th true leaf of No.44 than in its counterpart in SHF (Fig. 2D). Taken together, these results show that No.44 is resistant to ToLCNDV-ES not only at the young seedling stage but also at the fruit-setting stage.

**Table 2:** Evaluation of tomato leaf curl New Delhi virus (ToLCNDV) resistance in No.44 by viral graft transmission

Cucumber accession^v^	Number of plants			Disease severity index (DSI) score^z^			
				30 dpi		60 dpi	
	Inoculated^w^	Infected^x^	(%)^y^	5th leaf	10th leaf	9th leaf	20 leaf
SHF	5	5	100	3^a^	3.8^a^	4^a^	1.4^a^
44	5	5	100	1.1^b^	0^b^	1.4^b^	0^b^

^v^SHF = Sagami Hanjiro Fushinari (begomovirus susceptible cultivar)

^w^ToLCNDV-[ES-Alm-Cuc-16] was inoculated

^x^Virus infection was detected by PCR

^y^(Number of plants with infected / number of plants inoculated) × 100

^z^The DSI ranged from a score of 0 to 4 as follows: 0, no symptoms; 1, a few yellow spots on the leaf; 2, yellow spots around the whole leaf; 3, yellowing of the leaf with mild curling; 4, heavy yellowing of the leaf with curling. Different letters indicate significant differences between means (Mann-Whitney U test, *p* < 0.05)

### Genetic mapping of ToLCNDV-ES resistance in F_2_ populations

**Fig. 3:**
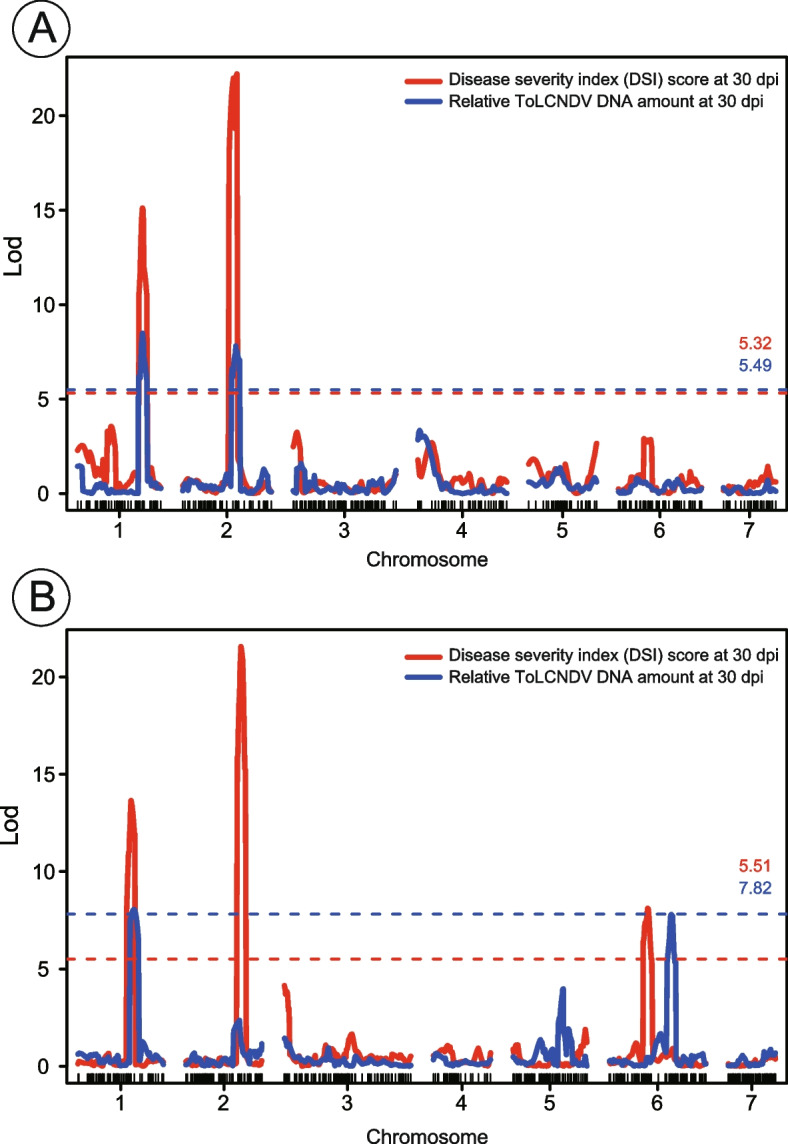
Linkage analysis of the logarithm of the odds (LOD) scores of ToLCNDV-ES resistance in F_2_ populations generated by crossing the begomovirus-susceptible cultivar ‘Sagami Hanjiro Fushinari’ (SHF; susceptible) and No.44 (resistant). The red line plotted indicates the LOD scores of the disease severity index (DSI) at 30 days post inoculation (dpi) and the blue line indicates the level of ToLCNDV-ES viral DNA accumulation at 30 dpi for **A** first round of the SHF × No.44 F_2_ population (*n* = 187) and **B** second round of SHF × No.44 F_2_ population (*n* = 143)

For the genetic mapping of ToLCNDV-ES resistance, two rounds of phenotyping were conducted with two independent F_2_ populations generated by crossing the begomovirus-susceptible cultivar SHF and No.44. In the first F_2_ population, 187 out of the 203 individuals agroinfiltrated with ToLCNDV-ES tested positive (92%) for virus infection at 30 dpi, as determined by PCR. These ToLCNDV-ES-positive plants were analyzed further. At 30 dpi, phenotypic segregations were as shown in Fig. S1A, and number of resistant individuals (DSI 0–1) to susceptible individuals (DSI 2–4) segregated into 1:3 for the F_2_ population (*n* = 187), as shown via the χ^2^ test, indicating that ToLCNDV-ES resistance was controlled by a single recessive gene (Table S1). Linkage analysis of ToLCNDV-ES resistance in the first round of the SHF × No.44 F_2_ population (*n* = 187) was performed using 309 SNPs discovered by restriction site-associated DNA sequencing (RAD-seq). The number of linkage groups was the same as the chromosome number of cucumber (*C. sativus*), and the total size of the linkage map was 647.8 cM (average marker distance = 2.1 cM). Two significant QTLs, one on chromosome 1 and the other on chromosome 2, were detected by a composite interval mapping (CIM) analysis of the SHF × No.44 F_2_ population; these QTLs were denoted as *cucumber yellow leaf curl disease resistance-1* (*Cy-1*) and *Cy-2*, respectively (Fig. 3A, Table 3). The QTLs explaining less than 10% of the phenotypic variation were defined as minor QTLs, whereas the QTLs explaining more than 10% of the phenotypic variation were defined as major QTLs according to previous report [48]. The highest peaks and narrowest intervals of QTLs were detected for the DSI score at 30 dpi. A major QTL *Cy-1* with logarithm of odds (LOD) score of 15.1 was detected at the physical position of 24,837,399 on chromosome 1 of the reference sequence (Chinese Long, ver. 3), and the other major QTL *Cy-2* (LOD score = 22.2) was detected at the physical position of 17,866,822 on chromosome 2. *Cy-1* and *Cy-2* explained 20.0% and 30.7% of the total phenotypic variation, respectively.

**Table 3:** Quantitative trait loci (QTL) identified in the F_2_ populations of SHF × No.44

Exp.	Trait	Chr	QTL	Contributing	LOD	PVE^x^ %	Peak		1.8-LOD support interval					
			name	parent			Marker	Position^y^	Left marker	Position^y^	Right marker	Position^y^	cM	Genes^z^
1	Symptoms 30 dpi	1	*Cy-1*	No.44	15.1	20.0	S1_24837399	24,837,399	S1_24327273	24,327,273	S1_25380586	25,380,586	71.4–75.8	148
1	Viral accumulation 30 dpi	1	*Cy-1*	No.44	9.2	16.9	S1_24837399	24,837,399	S1_24327273	24,327,273	S1_27446172	27,446,172	71.4–82.9	394
2	Symptoms 30 dpi	1	*Cy-1*	No.44	13.7	18.0	S1_24211834	24,211,834	S1_23709521	23,709,521	S1_26270945	26,270,945	56.7–68.9	346
2	Viral accumulation 30 dpi	1	*Cy-1*	No.44	8.1	21.1	S1_25309314	25,309,314	S1_23709521	23,709,521	S1_26270945	26,270,945	56.7–68.9	346
1	Symptoms 30 dpi	2	*cy-2*	No.44	22.2	30.7	S2_17866822	17,866,822	S2_17265106	17,265,106	S2_18252890	18,252,890	60.4–63.4	166
1	Viral accumulation 30 dpi	2	*cy-2*	No.44	10.5	12.7	S2_17323252	17,323,252	S2_14401361	14,401,361	S2_19006471	19,006,471	50.1–70.5	573
2	Symptoms 30 dpi	2	*cy-2*	No.44	21.6	36.3	S2_17695694	17,695,694	S2_17251044	17,251,044	S2_18251145	18,251,145	59.4–64.9	169
2	Symptoms 30 dpi	6	*Cy-6*	No.44	8.1	8.5	S6_11725560	11,725,560	S6_9555295	9,555,295	S6_13328338	13,328,338	36.1–46.3	432

^x^Percent of the phenotypic variation explained by the QTL

^y^Physical position in reference genome sequence of cucumber Chinese Long v3

^z^Number of potential candidate genes located in the 1.8-LOD interval of the QTL

In the second F_2_ population, 143 out of 149 individuals tested positive (96%) for ToLCNDV-ES infection at 30 dpi. Phenotypic segregations were as shown in Fig. S1B, and the segregation ratio indicated that ToLCNDV-ES resistance was conferred by a single recessive gene (Table S1). Linkage analysis of ToLCNDV-ES resistance was performed using 455 SNPs discovered by RAD-seq (*n* = 143). The number of linkage groups was the same as the chromosome number of cucumber, and the linkage map size was 623.3 cM (average marker distance = 2.0 cM). A CIM analysis of the SHF × No.44 F_2_ population detected three significant QTLs on chromosomes 1, 2, and 6 (Fig. 3B, Table 3). The highest peaks and narrowest intervals of QTLs were detected for the DSI score at 30 dpi. Among the three QTLs detected, two major QTLs had genomic regions that overlapped with *Cy-1* and *Cy-2* identified in the first round of QTL analysis. The *Cy-1* QTL (LOD score = 13.7) was detected at position 24,211,834 on chromosome 1, and the *Cy-2* QTL (LOD score = 21.6) was detected at position 17,695,694 on chromosome 2. *Cy-1* and *Cy-2* explained 18.0% and 36.3% of the total phenotypic variation, respectively. Additionally, the third minor QTL *Cy-6* (LOD score = 8.1) located at the physical position 11,725,560 on chromosome 6 explained 8.5% of the total phenotypic variation.

The major QTLs *Cy-1* and *Cy-2* that were consistently detected in the two rounds of genetic mapping were further analyzed. Individuals with different allelic combinations of *Cy-1* and *Cy-2* were identified from the first-round F_2_ population on the basis of the genotypes of the closest markers at the LOD peaks. Plants homozygous for the No.44 allele at marker S2_17866822 on chromosome 2 were more resistant to ToLCNDV-ES and had lower virus titers than plants heterozygous or homozygous for the SHF allele. These results indicated that *Cy-2* acts recessively to increase resistance to ToLCNDV-ES (Fig. S2A and B). In light of these results, this locus is hereinafter referred to as *cy-2*. In contrast, except for a single case, plants homozygous and heterozygous for the No.44 allele at marker S1_24837399 on chromosome 1 were more resistant to ToLCNDV-ES and had lower virus titers than those of plants homozygous for the SHF allele, indicating that *Cy-1* acts dominantly (Fig. S2A and B). In addition, epistatic interaction between *Cy-1* and *cy-2* was detected (*p* = 0.017) for viral DNA accumulation (Fig. S2B). The effect of the No.44 allele at *Cy-1* to increase resistance (i.e., decrease DSI score) was observed in two genotype classes, homozygous for the No.44 allele at the *cy-2* and heterozygous, but not in the class homozygous for the SHF allele at the *cy-2*. In the second-round F_2_ population, a similar dominant nature of *Cy-1* with the first-round F_2_ population was detected except for homozygous for the No.44 allele at the *cy-2* (Fig. S2C). No statistically significant epistatic interaction between *Cy-1* and *cy-2* was detected in this case (*p* = 0.460). The overlapping regions of *Cy-1* and *cy-2* identified in the two rounds of linkage analyses included 468 and 573 candidate genes, respectively (Table S2).

### Fine mapping of the ToLCNDV-ES resistance QTL *Cy-1*

**Fig. 4:**
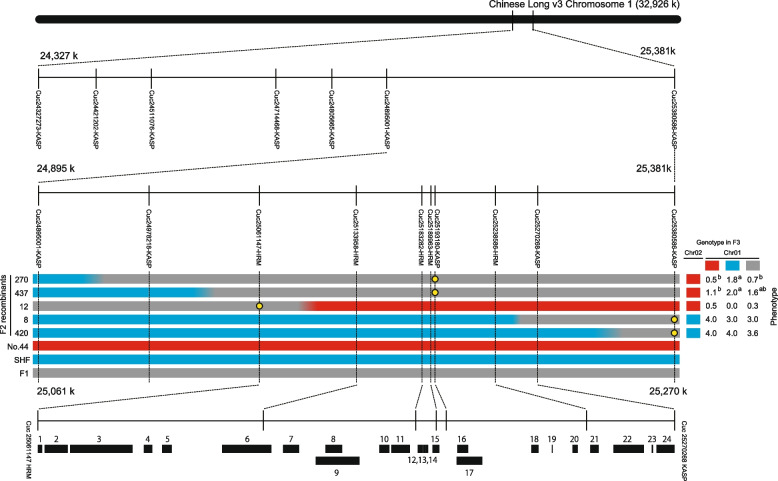
Fine mapping of candidate genes at *Cy-1* on chromosome 1. *Cy-1* was mapped between the Cuc25061147-HRM and Cuc25270268-KASP markers on chromosome 1. The red, blue, and gray bars indicate No.44 allele (homozygous dominant), SHF allele (homozygous recessive), and heterozygous genotype, respectively. F_3_ populations derived from the selfing of F_2_ recombinants (No.8, No.12, No.270, No.420, and No.437) were genotyped using one of three markers, namely, Cuc25061147-HRM, Cuc25193180-KASP, or Cuc25380586-KASP, as indicated by the yellow circle in the figure, to assess the genotype of the target region. On the right side of the figure, the genotype and ToLCNDV-ES resistance phenotype are indicated for F_3_ population: red, blue, and gray indicate No.44 allele (homozygous recessive), SHF allele (homozygous dominant), and heterozygous genotype, respectively. On the lower side of the figure, 24 predicted genes are indicated as black boxes. SHF indicates the begomovirus-susceptible cultivar ‘Sagami Hanjiro Fushinari’. Different letters indicate significant differences in the disease severity index (DSI) between the means of F_3_ individuals (Bonferroni–Dunn test; *p* < 0.05)

We further finely mapped the ToLCNDV-ES resistance QTL *Cy-1* (Fig. 4). Two KASP markers (Cuc24327273-KASP and Cuc25380586-KASP) were developed and five recombinants (No.8, No.12, No.270, No.420, and No.437) were screened from the newly prepared F_2_ population (*n* = 752) to narrow down the candidate region. The recombination points were verified using 13 additional markers, consisting of eight KASP and five high-resolution melting (HRM) markers. F_3_ populations were obtained by self-pollinating these F_2_ recombinants. The phenotypes of three F_3_ populations derived from the F_2_ individuals, No.270, No.437, and No.12, did not fit the genotypes of three markers, namely Cuc24895001-KASP, Cuc24978218-KASP, and Cuc25061147-HRM, respectively. While the phenotypes of two F_3_ populations derived from the F_2_ individuals, No.8 and No.420, did not fit the genotypes of two markers, namely Cuc25270268-KASP and Cuc25380586-KASP, respectively. From these results, the ToLCNDV-ES resistance QTL *Cy-1* was mapped to a 209-kb region between the Cuc25061147-HRM marker and the Cuc25270268-KASP marker. In the Chinese Long genome (ver.3), 24 putative genes were identified in this target region (Table 4). Whole-genome resequencing of the two parents revealed that, compared with those of SHF and Chinese Long, only two genes, CsaV3_1G039730 and CsaV3_1G039870, had nonsynonymous substitutions in their open reading frames (ORFs) in No.44. Furthermore, the transcript level of CsaV3_1G039730 was significantly greater in ToLCNDV-ES-infected and mock-inoculated No.44 than in ToLCNDV-ES-infected and mock-inoculated SHF plants, as determined by RNA-seq analysis (Table 4). On the basis of these results, CsaV3_1G039730, which encodes an RNA-dependent RNA polymerase (RDR), was identified as a potential candidate gene conferring resistance to ToLCNDV-ES within QTL *Cy-1* linked to ToLCNDV-ES resistance.

**Table 4:** Predicted ORFs located in the candidate region

ORF	ID^z^	Proteinsize	Description	Query coverage (%)	E value	Identity	GenBank ID	Re-seq^y^	RNA-seq (TPM)^x^			
									Mock		ToLCNDV	
									SHF	No.44	SHF	No.44
1	CsaV3_1G039680	260	Late embryogenesis abundant protein 47	99	0	100.0	XP_004135801	0	0.2	7.2*	0.4	5.3
2	CsaV3_1G039690	324	Cysteine synthase	99	0	100.0	XP_004135663	0	4.2	3.8	5.5	4.4
3	CsaV3_1G039700	325	Cysteine synthase	99	0	100.0	XP_011659947	0	82.9	62.1	39.6	46.8
4	CsaV3_1G039710	403	DNA-binding storekeeper protein-related transcriptional regulator	99	0	100.0	XP_004135666	0	66.0	63.1	80.2	47.3
5	CsaV3_1G039720	137	Guanine deaminase	99	2.00E-96	100.0	KAE8653398	0	67.3	32.0	81.5	39.1*
6	CsaV3_1G039730	980	RNA-dependent RNA polymerase	99	0	100.0	XP_031744299	7**	0.2	8.9*	0.4	9.0*
7	CsaV3_1G039740	592	Malic enzyme	99	0	100.0	XP_004135668	0	252.0	68.1*	55.4	38.7
8	CsaV3_1G039750	381	Protein-serine/threonine phosphatase	99	0	100.0	XP_004135669	0	22.5	57.1	34.6	49.3*
9	CsaV3_1G039760	1232	ABC transporter	99	0	100.0	XP_011659952	0	0.1	0.4	0.1	0.1
10	CsaV3_1G039770	377	Senescence/dehydration-associated protein	99	0	100.0	XP_004135671	0	3.1	1.5	1.7	1.2
11	CsaV3_1G039780	534	Ceramide glucosyltransferase	99	0	100.0	XP_004135670	0	15.7	2.9*	5.3	3.3
12	CsaV3_1G039790	530	SpoU_sub_bind domain-containing protein	99	0	100.0	XP_004135672	2	14.4	4.1*	1.5	2.6*
13	CsaV3_1G039800	58	Unknown protein	98	3.00E-33	100.0	KGN66177	0	43.4	27.3	9.9	86.5
14	CsaV3_1G039810	227	Mitochondrial import inner membrane translocase subunit TIM17-2-like	99	3.00E-159	100.0	XP_004135673	1	4.1	32.1*	2.0	115.7
15	CsaV3_1G039820	372	Receptor-like protein kinase HSL1	99	0	100.0	KGN66179	0	80.1	12.7	8.7	8.7
16	CsaV3_1G039830	499	Phosphotransferase	99	0	100.0	XP_004135675	0	8.2	40.3*	1.4	75.9
17	CsaV3_1G039840	997	Receptor-like protein kinase 5	99	0	100.0	XP_004135674	0	0.6	0.0	0.0	2.0
18	CsaV3_1G039850	539	Protein DETOXIFICATION	99	0	100.0	XP_011659955	0	3.9	1.7*	2.0	0.8
19	CsaV3_1G039860	61	Unknown protein	98	9.00E-34	100.0	KGN66183	0	0.2	0.1	0.1	0.3
20	CsaV3_1G039870	411	Protein IQ-DOMAIN 14-like	99	0	100.0	XP_011659956	1**	5.0	7.2	2.7	3.0
21	CsaV3_1G039880	387	Pectinesterase	99	0	100.0	XP_004135808	0	119.9	29.2	250.1	93.6
22	CsaV3_1G039890	1539	Non-specific serine/threonine protein kinase	99	0	100.0	XP_011659957	0	25.0	13.4	8.7	5.4
23	CsaV3_1G039900	70	Programmed cell death protein 4-like	98	9.00E-40	100.0	KAE8653402	0	34.9	60.6	28.5	52.1
24	CsaV3_1G039910	646	Arginyl-tRNA synthetase	99	0	100.0	XP_011659958	0	5.3	2.7*	3.6	0.7*

^z^ID for genes predicted in the reference genome of Chinese Long v3

^y^Predicted nonsynonymous substitution between No.44 and SHF from whole-genome resequencing. ** indicates predicted nonsynonymous substitution between No.44 and Chinese Long

^x^Average values for transcripts per kilobase million (TPM) of candidate ORFs. Three each for mock and ToLCNDV-infected individuals at 30 dpi were used for RNA-seq analysis. * indicate significant differences between means (Student’s t-test, *p* < 0.05)

### Analysis of *CsRDR3* encoding an RNA-dependent RNA polymerase

**Fig. 5:**
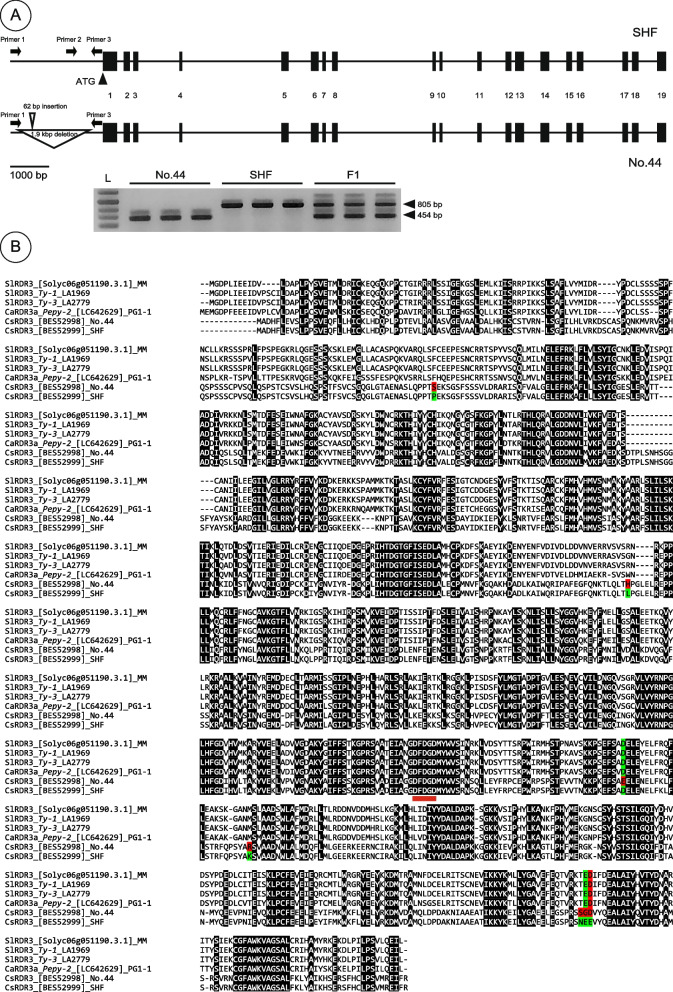
Molecular genetic identification of *CsRDR3*. **A** Gene structure of *CsRDR3* in ‘Sagami Hanjiro Fushinari’ (SHF; susceptible) and No.44 (resistant). Closed black boxes indicate exons identified from comparisons of the transcript and genome sequences of *CsRDR3*. In No.44, the promoter region of *CsRDR3* had a 1.9-kb deletion and 62-bp insertion. Agarose gel showing the PCR amplicons of *CsRDR3* dereived from No.44, SHF, and F_1_ plants using an Indel marker targeting the promoter region of *CsRDR3*. L indicates the 1-kb DNA ladder. Uncropped full length gel image is provided in the Supplementary Information as Supplementary Fig. S3. **B** Multiple sequence alignment of CsRDR3 in No.44 and SHF and its homologs SlRDR (*Solanum lycopersicum*) and CaRDR3a (*Capsicum annuum*) using Clustal Omega. The red underline represents the DFDGD motif, and the red colored letters indicate amino acid substitutions in No.44 compared with those in SHF

**Fig. 6:**
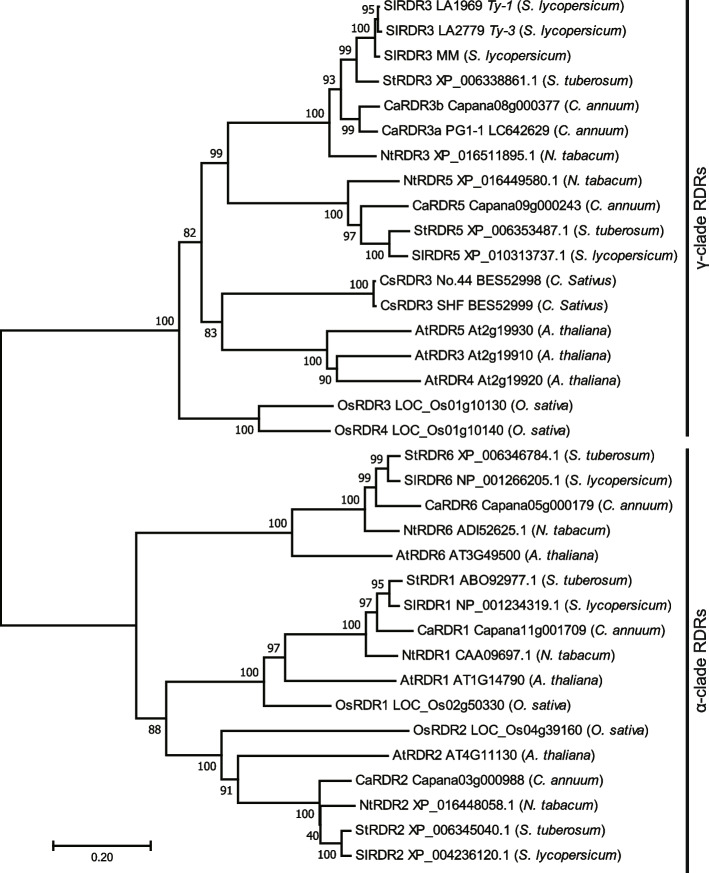
Neighbor-joining tree of the protein sequences of CsRDR3 and RDRs of other plant species. Bootstrap values were calculated based on 1000 replicates and are indicated at nodes

The full-length sequences of CsaV3_1G039730, *CsRDR3*, from No.44 and SHF were isolated by reverse-transcription PCR (RT‒PCR) and analyzed. These analyses revealed that *CsRDR3* consisted of 19 exons (Fig. 5A). CsRDR3 showed high sequence similarly with SlRDR3 (*Ty-1*/*Ty-3*) of tomato and CaRDR3a (*Pepy-2*) of capsicum (Fig. 5B). Seven nonsynonymous substitutions were detected in the amino acid sequence of CsRDR3 in No.44 compared with its counterpart in SHF (Fig. 5B). In addition, compared with those in SHF, an approximately 1.9-kb deletion and a 62-bp insertion were identified in the *CsRDR3* promoter region in No.44 (Fig. 5A). A BLAST analysis of this 1.9-kb fragment in the genomic DNA of SHF and Chinese Long revealed no similarity to any annotated sequences, including transposable elements. Multiplex PCR successfully amplified a 454-bp band from plants homozygous for the No.44-allele, an 805-bp band from plants homozygous for the SHF-allele, and 454-bp and 805-bp bands from heterozygous plants (Fig. 5A). This co-dominant DNA marker can be used for genotyping the resistant and susceptible alleles of *CsRDR3*. According to the results of the phylogenetic analysis, the RDRs from different plant species formed two clusters, namely α and γ -clades of RDR (Fig. 6). Phylogenetic analysis showed that the CsRDR3s of No.44 and SHF exhibited high amino acid sequence similarity with RDR3, RDR4, and RDR5 of *Arabidopsis thaliana*, which are γ-clade RDRs. The RDRs of tomato (SlRDR3), pepper (SlRDR3a), potato (StRDR3), and tobacco (NtRDR3) constituted an independent clade from the CsRDR3s within the γ-clade RDRs.

### Virus-induced gene silencing of *CsRDR3*

All the analyses so far support *CsRDR3* as a strong candidate for the gene conferring ToLCNDV-ES resistance at the QTL *Cy-1*. To further verify this hypothesis, we conducted virus induced gene silencing (VIGS) of *CsRDR3* to confirm its involvement in ToLCNDV-ES resistance. In an initial experiment in which *CsRDR3* was silenced in No.44 by VIGS, changes in resistance were not detected because of the effect of another major ToLCNDV-ES resistance QTL *cy-2* on chromosome 2. To address this problem, we used two chromosome segment substitution lines, No.148 and No.489, which were homozygous for the No.44 genotype at the target region on chromosome 1 and homozygous for the SHF genotype at the target region on chromosome 2. Moreover, we used *CsRDR3*-specific gene fragments as inserts for the apple latent spherical virus (ALSV) vector to avoid the off-target effects of VIGS.

**Fig. 7:**
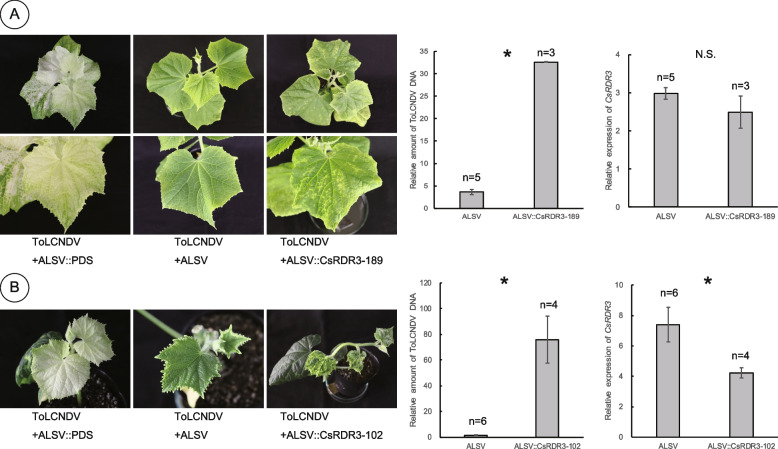
Functional analysis of *CsRDR3* by virus-induced gene silencing (VIGS). Effect of VIGS on ToLCNDV-ES caused symptoms, relative ToLCNDV-ES viral DNA accumulation levels, and *CsRDR3* transcript levels in **A** No.148 and **B** No.489 plants at 40 days post inoculation (dpi). Lines No.148 and No.489 were homozygous for the No.44 (resistant) genotype in the target region on chromosome 1 containing *Cy-1* and for the ‘Sagami Hanjiro Fushinari’ (SHF; susceptible) genotype in the target region on chromosome 2 containing *cy-2*. Young upper leaves of ToLCNDV-ES-infected plants were collected at 40 dpi for analysis of viral DNA accumulation by qPCR. Normalized relative viral DNA values were calculated using the 25S rRNA gene. The numbers of biological replicates are indicated on each bar. The data are presented as the mean ± standard error (SE). Significant differences between means (Student’s *t* test; *p* < 0.05) are indicated with asterisks and N.S. indicates not significant

For VIGS of *CsRDR3* in No.148, the ALSV construct pBICAL2::CsRDR3-189 was used. The characteristic photobleaching effect appeared in the topmost leaves after 28 dpi and was subsequently enhanced in the phytoene desaturase (PDS) gene-silenced No.148 plants (Fig. 7A). Plants with mixed ToLCNDV-ES and ALSV infections, as confirmed by PCR, were selected for further analysis. The control No.148 plants with mixed infection of ToLCNDV-ES and wild-type ALSV were resistant to ToLCNDV-ES. In contrast, the *CsRDR3*-silenced No.148 plants exhibited mild leaf-yellowing symptoms and accumulated nine times more ToLCNDV-ES viral DNA, which was significantly greater than that of the control plants. The transcript levels of *CsRDR3* tended to be lower in *CsRDR3*-silenced No.148 plants than in control plants at 40 dpi, but with no statistical differences. Although the first experiment partially supported our hypothesis that *CsRDR3* is involved in ToLCNDV-ES resistance, further experiments are needed.

Since the efficiency of VIGS by ALSV is reportedly affected by the genotype of the host plant and the size of the fragment inserted into the virus vector [49, 50], we used another chromosome segment substitution line, No.489, and ALSV construct pBICAL2::CsRDR3-102, which has a shorter fragment from a different part of the *CsRDR3* gene (Fig. 7B). The PDS gene-silenced No.489 plants started to show a photobleached phenotype in the developing leaves at 28 dpi. Since the growth of No.489 was more vigorous than that of No.148 under the same experimental conditions, we cut back the plants at 28 dpi and used the newly elongating lateral branches for analyses. At 40 dpi, the *PDS*-silenced No.489 plants exhibited a highly uniform photobleached phenotype. The control No.489 plants with mixed infection of ToLCNDV-ES and wild-type ALSV were resistant to ToLCNDV-ES. In contrast, the *CsRDR3*-silenced No.489 plants exhibited severe leaf curling and yellowing symptoms. In addition, there were 51-fold more viral DNA of ToLCNDV-ES accumulated in the *CsRDR3*-silenced No.489 plants than in the control plants. Moreover, the *CsRDR3* transcript level was significantly lower in the *CsRDR3*-silenced No.489 plants than in the control No.489 plants. We also performed VIGS for other candidate genes that had either nonsynonymous substitution in the ORF or difference in gene expression in ToLCNDV-ES-infected plants (Table 4), such as CsaV3_1G039720, CsaV3_1G039750, CsaV3_1G039790, CsaV3_1G039810, CsaV3_1G039870, and CsaV3_1G039910, but no loss-of-resistance phenotypes were observed in the gene-silenced plants (supplementary Fig. S4). In conclusion, that *CsRDR3* is most likely the gene underlying ToLCNDV-ES resistance at QTL *Cy-1* in No.44.

## Discussion

ToLCNDV was first isolated in India from diseased tomato plants [14]. Since then, its distribution has been reported from the Indian subcontinent, Southeast Asia, the Middle East, and the Mediterranean Basin [8]. The ToLCNDV isolates from Spain, Italy, Morocco, Algeria, and Tunisia in the Mediterranean Basin are monophyletic and form a nested clade within a much more diverse group of virus isolates from the Indian subcontinent. This strongly indicates that the ToLCNDV population in the Mediterranean Basin was founded by a single virus that originated from the Indian subcontinent [7, 8, 16]. A similar phylogenetic pattern was displayed for Southeast Asian ToLCNDV isolates from Thailand, Laos, Indonesia, and Taiwan. In the present study, we demonstrated that No.44 is resistant to the ToLCNDV-ES isolate from Spain and to the highly pathogenic Indonesian isolate ToLCNDV-IDN, which are the representative ToLCNDV isolates from the Mediterranean Basin and Southeast Asia, respectively.

High-throughput sequencing technology has enabled us to obtain DNA polymorphisms easily, even for non-model plant species. Meanwhile, successful genetic mapping of the target gene largely depends on accurate phenotyping of target traits which requires deliberate and laborious work but also needs improved operational efficiency. To take the balance between accuracy and efficiency, ToLCNDV-ES was inoculated to F_2_ individuals by agroinfiltration rather than grafting in our linkage analyses. Graft-inoculation of begomoviruses is a promising method to deliver virus to evaluating plant material as shown in Table 2 and our previous studies [32, 33, 58]. However, grafting requires laborious work and takes a relatively long time to obtain phenotype data compared to agroinfiltration. In our agroinfiltration experiments shown in Table 1, ToLCNDV-ES infected No.44 plants in 26–27%. However, ToLCNDV-ES successfully infected F_2_ populations in 92–96%. This may be partially due to the trained skill of the researcher through the experience of agroinfiltration, however, it was still inevitable for inoculation escapes, 6 out of 203 F_2_ individuals and 6 out of 149 F_2_ individuals in the first and second rounds of linkage analyses, which we excluded these individuals from our study through PCR-based diagnosis. These experimental procedures, visual-based 0–4 arbitrary symptom severity rating method for DSI score, and fluctuation of gene frequency in the seed bulk may have affected the segregation ratio either singly or in combination. It should be noted that two rounds of phenotyping with two independent F_2_ populations consistently mapped two major QTLs for ToLCNDV-ES resistance on chromosomes 1 and 2 in CIM analyses. Moreover, further fine-mapping using F_3_ populations narrowed the candidate region of chromosome 1 to a genomic fragment containing 24 genes. We cannot dismiss the possibility that the other 23 candidate genes, in addition to *RDR* in the target genomic region, also contributed to ToLCNDV-ES resistance in No.44 according to our forward genetic analysis. However, the results of whole-genome sequencing, RNA-seq analyses, and previous reports regarding begomovirus resistance in tomato and capsicum conferred by *Ty-1*/*Ty-3* and *Pepy-2* encoding RDRs [33, 35], strongly indicated that *RDR* is most likely the gene responsible for ToLCNDV resistance.

To strengthen the scientific basis of our hypothesis, we conducted reverse genetic analyses by using ALSV-based VIGS in cucumber. The efficiency of VIGS was greater in the second experiment in which chromosome segment substitution line No.489 and the ALSV construct pBICAL2::CsRDR3-102 were used than the first experiment, in which chromosome segment substitution line No.148 and the ALSV construct pBICAL2::CsRDR3-189 were used. There are several possible explanations for these results. Because it is reported that the effectiveness of ALSV-mediated VIGS is genotype dependent in soybean (*Glycine max*) [49], the difference in genotype between chromosome segment substitution cucumber line No.489 and No.148 may have affected the VIGS efficiency of *CsRDR3*. The length and/or position of inserted sequences are also reported to affect the efficiency of VIGS [50]. Since we used *CsRDR3* at different positions and fragment lengths for pBICAL2::CsRDR3-102 and pBICAL2::CsRDR3-189, this may have resulted in differences in VIGS efficiency between the two experiments. Notably, there was good correspondence between the VIGS efficiency of *CsRDR3* and the symptomology observed or the accumulating ToLCNDV-ES viral DNA. Moreover, because the VIGS of other candidate genes, such as CsaV3_1G039720, CsaV3_1G039750, CsaV3_1G039790, CsaV3_1G039810, CsaV3_1G039870, and CsaV3_1G039910, induced no loss of resistance, as did the *CsRDR3* (CsaV3_1G039730)-silenced plants, we concluded that *CsRDR3* is the gene responsible for ToLCNDV-ES resistance at the major QTL *Cy-1.*

Several begomovirus resistance genes have been cloned from Solanaceae plants using elaborate forward and reverse genetic methods. In tomato, the TYLCV resistance genes *Ty-1*/*Ty-3* encode RDRs, *Ty-2* encodes a nucleotide-binding leucine-rich repeat (NB-LRR) protein, and *ty-5* encodes Pelota [34–36]. More recently, we identified *pepy-1* encoding Pelota and *Pepy-2* encoding an RDR that confer resistance to bipartite begomoviruses in capsicum [32, 33]. The resistance gene *Ty-1*, which is widely used in the breeding of F_1_ hybrid cultivars of tomato plants, also confers resistance to the curtovirus, the leafhopper-transmitted beet curly top virus, suggesting that RDR-based resistance represents broad resistance to geminiviruses [51]. Previously conducted genetic mapping of ToLCNDV-ES resistance in cucumbers revealed QTLs in the overlapping genomic region of chromosome 1, as detected in our study [44–46]. These previous findings indicate that *Cy-1,* which harbors *CsRDR3,* confers resistance to ToLCNDV-ES in multiple cucumber resources, not only in No.44.

In plants, the long double-stranded RNA molecules are cleaved to produce siRNAs by RNA-silencing pathways. The RDR protein participates in these pathways as a part of the antiviral defense mechanism. RDRs share a special conserved RDR catalytic domain, and eukaryotic RDRs can be grouped into RDRα, RDRβ, and RDRγ [52]. Members of the RDRβ clade are not found in plants but are conserved mainly among fungi [53]. In the genome of *A. thaliana*, there are six RDRs which are grouped into the RDRα and RDRγ clades [54]. *Arabidopsis* α-type RDRs have the DLDGD motif of eukaryotic RDRs but are not essential for the production of viral siRNAs in geminivirus-infected *Arabidopsis* [54, 55]. The functions of the three RDRγs in *Arabidopsis* with a DFDGD amino acid motif in the catalytic domain have not yet been described. In contrast, the function of γ-type RDRs related to geminivirus resistance in *Solanaceae* is better understood. According to our phylogenetic analysis, the cucumber CsRDR3 was clustered with γ-type RDRs, specifically SlRDR3 (*Ty-1*/*Ty-3*) of tomato and CaRDR3a (*Pepy-2*) of capsicum. Furthermore, it was demonstrated that the replication of begomoviral DNA is restricted by RDR via a transcriptional gene silencing pathway in tomato plants harboring *Ty-1* [37]. We speculate that CsRDR3 confers ToLCNDV resistance via a similar mechanism in cucumber. Analyses of the transcript levels and sequence differences in *CsRDR3* between ToLCNDV-resistant No.44 and susceptible SHF revealed higher gene transcript levels in No.44, as well as multiple amino acid substitutions in its CsRDR3a protein. Similar observations have been reported for *Ty-1* and its encoded RDR [35, 56]. However, further research is needed to determine whether one or both of these differences in *CsRDR3* is required for conferring resistance. Since γ-type RDR was demonstrated to confer resistance to begomovirus not only in *Solanaceae* but also in cucumber, other vegetable crops in cucurbit may also harbor a resistance allele of γ-type RDR. From a practical viewpoint, the multiplex PCR-based system developed in this study to distinguish between resistant and susceptible alleles of *CsRDR3* will be practically beneficial for DNA marker-assisted breeding of ToLCNDV resistance in cucumber using No.44 as a resistant source.

In this study, our genetic mapping analyses revealed three QTLs for ToLCNDV-ES resistance on chromosomes 1, 2, and 6 in the cucumber genome. However, the minor QTL on chromosome 6 was detected only in the second-round mapping population, and its effect on resistance was rather small. Moreover, *cy-2* on chromosome 2 was identified as a major QTL conferring resistance to ToLCNDV-ES. The *ty-5* and *pepy-1* genes, both of which encode Pelota, are well-known recessive genes involved in resistance to begomoviruses [32, 34]. No Pelota-encoding gene was present in the target region of *cy-2*, suggesting that this region contains a novel recessive resistance gene that is potentially useful as a plant disease susceptibility (*S*) gene. This gene could be the target of genome editing or artificial mutant screening for the application of resistance mechanisms to other crop species. Fine mapping and reverse genetic analysis of *cy-2* are now in progress. These analyses will aid in DNA marker-assisted breeding for durable and wide-spectrum begomovirus resistance when combined with *Cy-1* and will provide a theoretical basis for the practical application of this resistance mechanism in various crops.

## Conclusions

We conducted fine-mapping of a major QTL *Cy-1* on chromosome 1 for ToLCNDV-ES resistance in cucumber accession No.44. Among 24 genes located on the candidate region, *CsRDR3*, an ortholog of *Ty-1*/*Ty-3* of tomato and *Pepy-2* of capsicum, was found to be a strong candidate conferring ToLCNDV-ES resistance. Reverse genetic analysis of *CsRDR3* by VIGS also supported that this gene is responsible for ToLCNDV-ES resistance*.* DNA marker developed in this study to distinguish the resistant and susceptible alleles of *CsRDR3* will be practically beneficial for marker-assisted breeding of ToLCNDV resistance in cucumber using No.44 as a resistant source.

## Methods

### Plant material

The cucumber (*C. sativus*) accession No.44 originating from South Asia, which is resistant to ToLCNDV, and the susceptible cultivars ‘Natsu Suzumi’ (NS) (Takii seeds, Kyoto, Kyoto, Japan) and ‘Sagami Hanjiro Fushinari’ (SHF) were used in this study. The F_2_ and F_3_ populations generated by crossing SHF and No.44 were used for genetic mapping. Plants were cultivated in a growth room with loose temperature control (25–30 °C day, 23–25 °C night) and a photoperiod of 13-h light/11-h dark.

### ToLCNDV inoculation and detection

Two ToLCNDV isolates were used in this study: the ToLCNDV-ES isolate ES-Alm-Cuc-16 (GenBank accession numbers for DNA-A: LC596380, DNA-B: LC596383) from Spain and the ToLCNDV-IDN isolate BACu-20 (DNA-A: LC511775, DNA-B: LC511780) from Indonesia [15, 57]. Full details about the infectious clones and the inoculation method have been reported elsewhere [15, 58]. In brief, agrobacteria bearing the plasmids pGreenII-p35S-ToLCNDV-[ES-Alm-Cuc-16]-DNA-A and pGreenII-p35S-ToLCNDV-[ES-Alm-Cuc-16]-DNA-B or pGreenII-p35S-ToLCNDV-[BACu-20]-DNA-A and pGreenII-p35S-ToLCNDV-[BACu-20]-DNA-B were cultured to an optical density of 0.3 and subsequently used to agroinfiltrate cotyledons of No.44, SHF, NS, and SHF × No.44 F_1_, F_2_, and F_3_ plants before the first true leaf developed. The disease symptoms of each plant were scored at 15 and/or 30 dpi as the disease severity index (DSI). The DSI ranged from 0 to 4 and was scored as follows: 0, no symptoms; 1, a few yellow spots on the leaf; 2, yellow spots across the whole leaf; 3, yellowing of the leaf with mild curling; and 4, intense yellowing and curling of the leaf. For DNA and RNA extraction, the young upper leaves were collected and stored at − 80 °C. Statistical analyses for DSI scores were conducted using the non-parametric Bonferroni–Dunn test or Mann–Whitney U test with Excel Toukei ver. 7.0, and a *p* value less than 0.05 was considered statistically significant.

An inoculation experiment was conducted to evaluate the graft transmission of ToLCNDV-ES to No.44 and SHF plants. At approximately 30 dpi, ToLCNDV-ES-infected symptomatic NS plants were used as a scion and grafted onto uninoculated No.44 and SHF plants (as rootstocks), and the newly elongated lateral branches from the rootstock were evaluated for ToLCNDV-ES resistance. Plants were grown in a greenhouse. Disease symptoms were surveyed at 30 and 60 days after grafting. The fifth and 10th true leaves were collected at 30 days after grafting, and the ninth and 20th true leaves were collected at 60 days after grafting for DNA extraction.

A Nucleon PhytoPure Kit (GE Healthcare, Little Chalfont, Buckinghamshire, UK) was used to extract DNA from cucumber leaves. Conventional PCR and qPCR were conducted to detect and quantify the viral DNA of ToLCNDV following the methods of [59]. The primer sequences and PCR conditions used in these analyses are listed in Supplementary Tables S3 and S4. Statistical analysis for accumulating viral DNA amount were performed by Student’s *t* test in Excel Toukei ver. 7.0 and a *p* value less than 0.05 was considered statistically significant.

### QTL mapping of ToLCNDV resistance

In this study, two rounds of phenotyping were conducted for two independent F_2_ populations consisting of 187 and 143 individuals infected with ToLCNDV-ES, and the extracted DNA was subjected to the first and the second rounds of RAD-seq analysis. The RAD-seq libraries were created for F_2_ individuals and their parents and sequenced with the NovaSeq 6000 platform (Illumina, Hercules, CA, USA) following the methods of [60]. The obtained reads were trimmed and mapped to the genome sequence of Chinese Long, ver.3 (*C. sativus*), one of the latest cucumber genome sequences [61]. The precise methodology used for the data analysis is described elsewhere [32]. The genetic linkage maps were constructed from the single nucleotide polymorphism (SNP) RAD tags, and R/qtl was used to conduct QTL analyses by composite interval mapping (CIM) [62].

### Fine mapping

The NovaSeq 6000 platform (Illumina) was used for whole-genome resequencing of No.44 and SHF, and the data analysis of the obtained reads was conducted following the methods reported in [32]. Additional SNP markers were developed for the KBiosciences KASPar assay (LGC Genomics GmbH, Berlin, Germany) to further narrow the region of the QTL, and recombinants were screened from F_2_ individuals. Five F_2_ recombinants, namely, No.8, No.12, No.270, No.420, and No.437, were screened, and the additional KASP markers and high-resolution melting (HRM) markers were used to identify the recombination points in each F_2_ individual. HRM analysis was conducted following the methods reported in [32]. The primer sequences and PCR conditions used for fine mapping is listed in Supplementary Table S5 and Table S4. The recombinants were self-pollinated to obtain F_3_ populations.

### Analyses of the candidate gene

Sepasol-RNA I Super G extraction buffer (Nacalai Tesque, Kyoto, Japan) and High-Salt Solution for Precipitation (Plant) (Takara Bio, Shiga, Japan) were used to extract and purify the RNA from leaves collected at 30 dpi. Transcriptome profiling of three biological replicates for each treatment was conducted via RNA-seq using the NovaSeq 6000 platform (Illumina) for mock-inoculated and ToLCNDV-ES-infected No.44 and SHF plants according to the previously reported methods [33]. De novo assembly of reads obtained by RNA-seq was conducted by Trinity (v.2.8.3.) [63]. The ORF sequences of the candidate gene, *CsRDR3*, were amplified by RT‒PCR to confirm the result of De novo assembly. RT‒PCR was conducted following the previously reported methods using the cDNA template, KOD-plus Neo (Toyobo, Osaka, Japan), and the *CsRDR3*-specific primer pairs to amplify the ORF of the candidate gene [32]. The amplified PCR products were cloned into the TOPO vector (Thermo Fisher Scientific, MA, USA) and used for subsequent sequencing. Supplementary Table S3 contains the list of the sequences of the primers used for PCR. Multiple sequence comparisons of the predicted RDR amino acid sequences of *A. thaliana*, *Oryza sativa* (rice), *Nicotiana tabacum*, *S. tuberosum*, *S. lycopersicum*, and *C. annuum* were conducted using the MUSCLE program [64]. MEGA 7.0 was used to construct the phylogenetic tree via the neighbor‒joining method, with 1,000 bootstrap replicates [65].

### Reverse genetic analysis by virus-induced gene silencing

Apple latent spherical virus (ALSV) vectors were used for VIGS [50, 66]. In brief, partial coding sequences of *CsPDS* (102 bp), *CsRDR3* (CsaV3_1G039730) (189 bp) or *CsRDR3* (CsaV3_1G039730) (102 bp) were amplified from No.44 with the CsPDS-102-Xho/Bam, CsRDR3-189-Xho/Bam or CsRDR3-102-Xho/Bam primers, respectively (Supplementary Table S3). The obtained amplicons digested with *Xho*I and *Bam*HI were ligated with *Xho*I- and *Bam*HI-digested pBICAL2 to construct pBICAL2::CsPDS, pBICAL2::CsRDR3-189, and pBICAL2::CsRDR3-102. To avoid the effects of off-target genes, a BLASTn analysis was conducted using PCR fragment sequences as queries to design the gene-specific primers for each gene.

For VIGS, we used two chromosome segment substitution lines, No.148 and No.489, which were homozygous for the No.44 genotype at the target region on chromosome 1 and homozygous for the SHF genotype at the target region on chromosome 2. Cultures of *Agrobacterium* containing pBICAL1 and pBICAL2, pBICAL2::CsPDS, pBICAL2::CsRDR3-189, or pBICAL2::CsRDR3-102 were mixed at a ratio of 1:1 for agroinfiltration. Before the first true leaf developed, cotyledons were agroinfiltrated with the bacterial suspension at an optical density of 0.3. One day after agroinfiltration of ALSV, ToLCNDV-ES was agroinoculated following the methods reported previously [15, 67]. For agroinoculation, a colony inoculation procedure was performed on the hypocotyl just below the apex of the seedlings using *Agrobacterium* transformed with pGreenII-p35S-ToLCNDV-[ES-Alm-Cuc-16]-DNA-A + B. No.489 plants were cut back at 28 dpi to allow for elongation of lateral branches, which were subsequently used for analysis. Symptom survey and collection of young upper leaves were conducted at 40 dpi.

The viral DNA-A of ToLCNDV-ES was detected by conventional PCR and quantified by qPCR. ALSV infection was confirmed by conventional PCR using the ALSV-F/R primer pair (Table S3). The transcript levels of *CsRDR3* were analyzed via qRT‒PCR following the previously reported methods [32]. The primer sequences and PCR conditions used for real-time qRT‒PCR analyses are listed in Supplementary Tables S3 and S4. The *CsActin* reference gene was used to normalize the transcript level of the candidate gene, and 2^−ΔΔCt^ method was applied to calculate relative gene transcript levels. Statistical analyses for gene expression and viral DNA accumulation were conducted using Student’s *t* test, and a *p* value less than 0.05 was considered statistically significant.

## Supplementary Information

Supplementary Material 1.

Supplementary Material 2.

Supplementary Material 3.

Supplementary Material 4.

Supplementary Material 5.

Supplementary Material 6.

Supplementary Material 7.

Supplementary Material 8.

Supplementary Material 9.

## Data Availability

Data is provided within the manuscript or supplementary information files. Transcript sequences of *CsRDR3* for No.44 and SHF are submitted to the DNA Data Bank of Japan (https://www.ddbj.nig.ac.jp/index-e.html) with the following accession numbers LC778230 and LC778231. Further data is available from the authors upon reasonable request.

## References

[1] Che G, Zhang X. Molecular basis of cucumber fruit domestication. Curr Opin Plant Biol. 2019;47:38–46. 30253288 10.1016/j.pbi.2018.08.006

[2] Sebastian P, Schaefer H, Telford IR, Renner SS. Cucumber (*Cucumis sativus*) and melon (*C. melo*) have numerous wild relatives in Asia and Australia, and the sister species of melon is from Australia. Proc Natl Acad Sci U S A. 2010;107:14269–73. 20656934 10.1073/pnas.1005338107 PMC2922565

[3] Food and Agriculture Organization of the United Nations (FAOSTAT). Crop and livestock products: cucumbers and gherkins. 2021. https://www.fao.org/faostat/en/#data/QCL.

[4] International Committee on Taxonomy of Viruses (ICTV). 2020. https://talk.ictvonline.org/taxonomy/. Accessed 6 Sep 2023.

[5] Zhou X. Advances in understanding begomovirus satellites. Annu Rev Phytopathol. 2013;51:357–81. 23915133 10.1146/annurev-phyto-082712-102234

[6] Hanley-Bowdoin L, Bejarano ER, Robertson D, Mansoor S. Geminiviruses: masters at redirecting and reprogramming plant processes. Nat Rev Microbiol. 2013;11:777–88. 24100361 10.1038/nrmicro3117

[7] Moriones E, Praveen S, Chakraborty S. Tomato leaf curl New Delhi virus: an emerging virus complex threatening vegetable and fiber crops. Viruses. 2017;9: 264. 28934148 10.3390/v9100264 PMC5691616

[8] Zaidi SS, Martin DP, Amin I, Farooq M, Mansoor S. *Tomato leaf curl New Delhi virus*: a widespread bipartite begomovirus in the territory of monopartite begomoviruses. Mol Plant Pathol. 2017;18:901–11. 27553982 10.1111/mpp.12481 PMC6638225

[9] Chang H, Ku H, Tsai W, Chien R, Jan F. Identification and characterization of a mechanical transmissible begomovirus causing leaf curl on oriental melon. Eur J Plant Pathol. 2010;127:219–28.

[10] Hussain M, Manssor S, Iram S, Zafar Y, Briddon RW. First report of *tomato** leaf curl New Delhi virus* affecting chilli pepper in Pakistan. Plant Pathol. 2004;53:794.

[11] Ito T, Sharma P, Kittipakorn K, Ikegami M. Complete nucleotide sequence of a new isolate of tomato leaf curl New Delhi virus infecting cucumber, bottle gourd and muskmelon in Thailand. Arch Virol. 2008;153:611–3. 18193155 10.1007/s00705-007-0029-y

[12] Maruthi MN, Rekha AR, Cork A, et al. First report of *tom**ato leaf curl New Delhi virus* infecting tomato in Bangladesh. Plant Dis. 2005;89:1011. 30786642 10.1094/PD-89-1011C

[13] Mizutani T, Daryono BS, Ikegami M, Natsuaki KT. First report of *tom**ato leaf curl New Delhi virus* infecting cucumber central Java, Indonesia. Plant Dis. 2011;95:1485. 30731770 10.1094/PDIS-03-11-0196

[14] Padidam M, Beachy RN, Fauquet CM. Tomato leaf curl geminivirus from India has a bipartite genome and coat protein is not essential for infectivity. J Gen Virol. 1995;76:25–35. 7844539 10.1099/0022-1317-76-1-25

[15] Yamamoto H, Wakita Y, Kitaoka T, Fujishiro K, Kesumawati E, Koeda S. Southeast Asian isolate of the tomato leaf curl New Delhi virus shows higher pathogenicity against tomato and cucurbit crops compared to that of the Mediterranean isolate. Hort J. 2021;90:314–25.

[16] Nagendran K, Mohankumar S, Mohammed Faisal P, Bagewadi B, Karthikeyan G. Molecular evidence for the occurrence of the tomato leaf curl New Delhi virus on chayote (*Sechium edule*) in southern India. Virus Dis. 2017;28:425–9. 10.1007/s13337-017-0403-7 PMC5747844 29291235

[17] Sohrab SS, Mandal B, Pant RP, Varma A. First report of assosciation of tomato leaf curl virus-New Delhi with yellow mosaic disease of *Luffa cylindrica* in India. Plant Dis. 2003;87:1148. 30812834 10.1094/PDIS.2003.87.9.1148A

[18] Phaneendra C, Rao KR, Jain RK, Mandal B. is associated with pumpkin leaf curl: a new disease in Northern India. Ind J Virol. 2012;23:42–5. 10.1007/s13337-011-0054-z PMC3550806 23730002

[19] European and Mediterranean Plant Protection Organization (EPPO). 2022. https://gd.eppo.int/taxon/TOLCND. Accessed 6 Sep 2023.

[20] Fidan H, Yildiz K, Sarikaya P, Calis O. First report of tomato leaf curl New Delhi virus in Türkiye. New Dis Rep. 2023;47: e12180.

[21] Juárez M, Tovar R, Fiallo-Olivé E, et al. First detection of tomato leaf curl New Delhi virus infecting zucchini in Spain. Plant Dis. 2014;98:857. 30708660 10.1094/PDIS-10-13-1050-PDN

[22] Kheireddine A, Sifres A, Sáez C, Picó B, López C. First report of *to**mato leaf curl New Delhi virus* infecting cucurbit plants in Algeria. Plant Dis. 2019;103:3291.

[23] Mnari-Hattab M, Zammouri S, Belkadhi MS, et al. First report of *tomat**o leaf curl New Delhi virus* infecting cucurbits in Tunisia. New Dis Rep. 2015;31:21.

[24] Orfanidou CG, Malandraki L, Beris D, et al. First report of tomato lead curl New Delhi virus in zucchini crops in Greece. J Plant Pathol. 2019;101:799.

[25] Panno S, Iacono G, Davino M, et al. First report of tomato leaf curl New Delhi virus affecting zucchini squash in an important horticultural area of southern Italy. New Dis Rep. 2016;33:6.

[26] Ruiz ML, Simón A, Velasco L, García MC, Janssen D. First report of *toma**to leaf curl New Delhi virus* infecting tomato in Spain. Plant Dis. 2015;99:894.

[27] Sifres A, Sáez C, Ferriol M, et al. First report of *toma**to leaf curl New Delhi virus* infecting zucchini in Morocco. Plant Dis. 2018;102:1045.

[28] Yazdani-Khameneh S, Aboutorabi S, Shoori M, et al. Natural occurrence of *tom**ato leaf curl New Delhi virus* in Iranian cucurbit crops. Plant Pathol J. 2016;32:201–8. 27298595 10.5423/PPJ.OA.10.2015.0210 PMC4892816

[29] Gilbertson RL, Batuman O, Webster CG, Adkins S. Role of the insect supervectors *Bemisia tabaci* and *Frankliniella occidentalis* in the emergence and global spread of plant viruses. Annu Rev Virol. 2015;2:67–93. 26958907 10.1146/annurev-virology-031413-085410

[30] Palumbo JC, Horowitz AR, Prabhaker N. Insecticidal control and resistance management for *Bemisia tabaci*. Crop Prot. 2001;20:739–65.

[31] Rojas MR, Macedo MA, Maliano MR, et al. World management of geminiviruses. Annu Rev Phytopathol. 2018;56:637–77. 30149794 10.1146/annurev-phyto-080615-100327

[32] Koeda S, Onouchi M, Mori N, Pohan NS, Nagano AJ, Kesumawati E. A recessive gene *pepy-1* encoding Pelota confers resistance to begomovirus isolates of PepYLCIV and PepYLCAV in *Capsicum annuum*. Theor Appl Genet. 2021;134:2947–64. 34081151 10.1007/s00122-021-03870-7

[33] Koeda S, Mori N, Horiuchi R, Watanabe C, Nagano AJ, Shiragane H. PepYLCIV and PepYLCAV resistance gene *Pepy-2* encodes DFDGD-Class RNA-dependent RNA polymerase in *Capsicum*. Theor Appl Genet. 2022;135:2437–52. 35652932 10.1007/s00122-022-04125-9

[34] Lapidot M, Karniel U, Gelbart D, et al. A novel route controlling begomovirus resistance by the messenger RNA surveillance factor Pelota. PLoS Genet. 2015;11: e1005538. 26448569 10.1371/journal.pgen.1005538 PMC4598160

[35] Verlaan MG, Hutton SF, Ibrahem RM, et al. The tomato yellow leaf curl virus resistance genes *Ty-1* and *Ty-3* are allelic and code for DFDGD-class RNA-dependent RNA polymerases. PLoS Genet. 2013;9: e1003399. 23555305 10.1371/journal.pgen.1003399 PMC3610679

[36] Yamaguchi H, Ohnishi J, Saito A, et al. An NB-LRR gene, *TYNBS1*, is responsible for resistance mediated by the *Ty-2 Begomovirus* resistance locus of tomato. Theor Appl Genet. 2018;131:1345–62. 29532116 10.1007/s00122-018-3082-x

[37] Butterbach P, Verlaan MG, Dullemans A, et al. Tomato yellow leaf curl virus resistance by *Ty-1* involves increased cytosine methylation of viral genomes and is compromised by cucumber mosaic virus infection. Proc Natl Acad Sci U S A. 2014;111:12942–7. 25136118 10.1073/pnas.1400894111 PMC4156758

[38] Koeda S, Fujiwara I, Oka Y, Kesumawati E, Zakaria S, Kanzaki S. *Ty-2* and *Ty-3a* conferred resistance are insufficient against tomato yellow leaf curl Kanchanaburi virus from Southeast Asia in single or mixed infections of tomato. Plant Dis. 2020;104:3221–9. 33044916 10.1094/PDIS-03-20-0613-RE

[39] Lapidot M, Legg JP, Wintermantel WM, Polston JE. Management of whitefly-transmitted viruses in open-field production systems. Adv Virus Res. 2014;90:147–206. 25410102 10.1016/B978-0-12-801246-8.00003-2

[40] Islam S, Mushi AD, Mandal B, Kumar R, Behera TK. Genetics of resistance in Luffa cylindrica Roem. against tomato leaf curl New Delhi virus. Euphytica. 2010;174:83–9.

[41] López C, Ferriol M, Picó MB. Mechanical transmission of *tom**ato leaf curl New Delhi virus* to cucurbit germplasm: selection of tolerance sources in *Cucumis melo*. Euphytica. 2015;204:679–91.

[42] Sáez C, Matinez C, Ferriol M, et al. Resistance to tomato leaf curl New Delhi virus in *Cucurbita* spp. Ann Appl Biol. 2016;169:91–105.

[43] Romay G, Pitrat M, Lecoq H, et al. Resistance against melon chlorotic mosaic virus and tomato leaf curl New Delhi virus in melon. Plant Dis. 2019;103:2913–9. 31436474 10.1094/PDIS-02-19-0298-RE

[44] Bracuto V, Koeken AC, Muller F, Villada ES. Begomovirus resistance related genes. 2021;WO2021064118.

[45] Liberti D, Koelewijn HP, Driedonks N, Cangal G, Chynoweth R. Introgression of ToLCNDV-ES resistance conferring QTLs in *Cucumis sativus* plants. 2022;WO2022223550.

[46] Paz Z, Yogev O, Rodriguez Medina A. Tolerance to ToLCNDV in cucumber. 2021;WO2021019069.

[47] Sáez C, Ambrosio LGM, Miguel SM, et al. Resistant sources and genetic control of resistance to ToLCNDV in cucumber. Microorganisms. 2021;9: 913. 33923281 10.3390/microorganisms9050913 PMC8146778

[48] Collard BCY, Jahufer MZZ, Brouwer JB, Pang ECK. An introduction to markers, quantitative trait loci (QTL) mapping and marker-assisted selection for crop improvement: the basic concepts. Euphytica. 2005;142:169–96.

[49] Gedling CR, Ali EM, Gunadi A, et al. Improved apple latent spherical virus-induced gene silencing in multiple soybean genotypes through direct inoculation of agro-infiltrated *Nicotiana benthamiana* extract. Plant Methods. 2018;14:19. 29527233 10.1186/s13007-018-0286-7 PMC5838930

[50] Igarashi A, Yamagata K, Sugai T, et al. *Apple latent spherical virus* vectors for reliable and effective virus-induced gene silencing among a broad range of plants including tobacco, tomato, *Arabidopsis thaliana*, cucurbits, and legumes. Virology. 2009;386:407–16. 19243807 10.1016/j.virol.2009.01.039

[51] Voorburg CM, Yan Z, Bergua-Vidal M, Wolters AA, Bai Y, Kormelink R. *Ty-1*, a universal resistance gene against geminiviruses that is compromised by co-replication of a betasatellite. Mol Plant Pathol. 2020;21:160–72. 31756021 10.1111/mpp.12885 PMC6988424

[52] Willmann MR, Endres MW, Cook RT, Gregory BD. The functions of RNA-dependent RNA polymerases in Arabidopsis. Arabidopsis Book. 2011;9: e0146. 22303271 10.1199/tab.0146 PMC3268507

[53] Zhang DX, Spiering MJ, Nuss DL. Characterizing the roles of *Cryphonectria parasitica* RNA-dependent RNA polymerase-like genes in antiviral defense, viral recombination and transposon transcript accumulation. PLoS One. 2014;9: e108653. 25268858 10.1371/journal.pone.0108653 PMC4182546

[54] Wassenegger M, Krczal G. Nomenclature and functions of RNA-directed RNA polymerases. Trends Plant Sci. 2006;11:142–51. 16473542 10.1016/j.tplants.2006.01.003

[55] Aregger M, Borah BK, Seguin J, et al. Primary and secondary siRNAs in geminivirus-induced gene silencing. PLoS Pathog. 2012;8: e1002941. 23028332 10.1371/journal.ppat.1002941 PMC3460622

[56] Caro M, Verlaan MG, Julián O, et al. Assessing the genetic variation of *Ty-1* and *Ty-3* alleles conferring resistance to tomato yellow leaf curl virus in a broad tomato germplasm. Mol Breed. 2015;35:132. 26028987 10.1007/s11032-015-0329-y PMC4442973

[57] Kesumawati E, Okabe S, Khalil M, Alfan G, Bahagia P, Pohan N, Zakaria S, Koeda S. Molecular characterization of begomoviruses associated with yellow leaf curl disease in *Solanaceae* and *Cucurbitaceae* crops from Northern Sumatra, Indonesia. Hort J. 2020;89:410–6.

[58] Koeda S, Homma K, Tanaka Y, Onizaki D, Kesumawati E, Zakaria S, Kanzaki S. Inoculation of capsicums with *Pepper yellow leaf curl Indonesia virus* by combining agroinoculation and grafting. Hort J. 2018;87:364–71.

[59] Koeda S, Kitawaki A. Breakdown of *Ty-1*-based resistance to tomato yellow leaf curl virus in tomato plants at high temperatures. Phytopathology. 2024;114:294–303. 37321561 10.1094/PHYTO-04-23-0119-R

[60] Koeda S, Sato K, Saito H, Nagano AJ, Yasugi M, Kudoh H, Tanaka Y. Mutation in the putative ketoacyl-ACP reductase *CaKR1* induces loss of pungency in *Capsicum*. Theor Appl Genet. 2019;132:65–80. 30267113 10.1007/s00122-018-3195-2

[61] Li Q, Li H, Huang W, et al. A chromosome-scale genome assembly of cucumber (Cucumis sativus L.). Gigascience. 2019;8: giz072. 31216035 10.1093/gigascience/giz072 PMC6582320

[62] Broman KW, Wu H, Sen Ś, Churchill GA. R/qtl: QTL mapping in experimental crosses. Bioinformatics. 2003;19:889–90. 12724300 10.1093/bioinformatics/btg112

[63] Grabherr M, Haas B, Yassour M, et al. Full-length transcriptome assembly from RNA-Seq data without a reference genome. Nat Biotechnol. 2011;29:644–52. 21572440 10.1038/nbt.1883 PMC3571712

[64] Edgar RC. MUSCLE: multiple sequence alignment with high accuracy and high throughput. Nucleic Acids Res. 2004;32:1792–7. 15034147 10.1093/nar/gkh340 PMC390337

[65] Kumar S, Stecher G, Tamura K. MEGA7: Molecular evolutionary genetics analysis version 7.0 for bigger datasets. Mol Biol Evol. 2016;33:1870–4. 27004904 10.1093/molbev/msw054 PMC8210823

[66] Kawai T, Gonoi A, Nitta M, et al. Virus-induced gene silencing in apricot (*Prunus armeniaca* L.) and Japanese apricot (*P. mume* Siebold & Zucc.) with the *Apple latent spherical virus* vector system. J Jpn Soc Hortic Sci. 2014;83:23–31.

[67] Koeda S, Homma K, Tanaka Y, Kesumawati E, Zakaria S, Kanzaki S. Highly efficient agroinoculation method for tomato plants with *tom**ato yellow leaf curl Kanchanaburi virus*. Hort J. 2017;86:479–86.

