# Supplementary material for: Cy-1, a major QTL for tomato leaf curl New Delhi virus resistance, harbors a gene encoding a DFDGD-Class RNA-dependent RNA polymerase in cucumber (Cucumis sativus)
Source: BMC Plant Biol. 2024 Oct 2;24:879. doi: 10.1186/s12870-024-05591-7 (PMC11446051; doi:10.1186/s12870-024-05591-7)
Supplement: Supplementary file 1 — Supplementary Material 1. [file 12870_2024_5591_MOESM1_ESM.pdf]

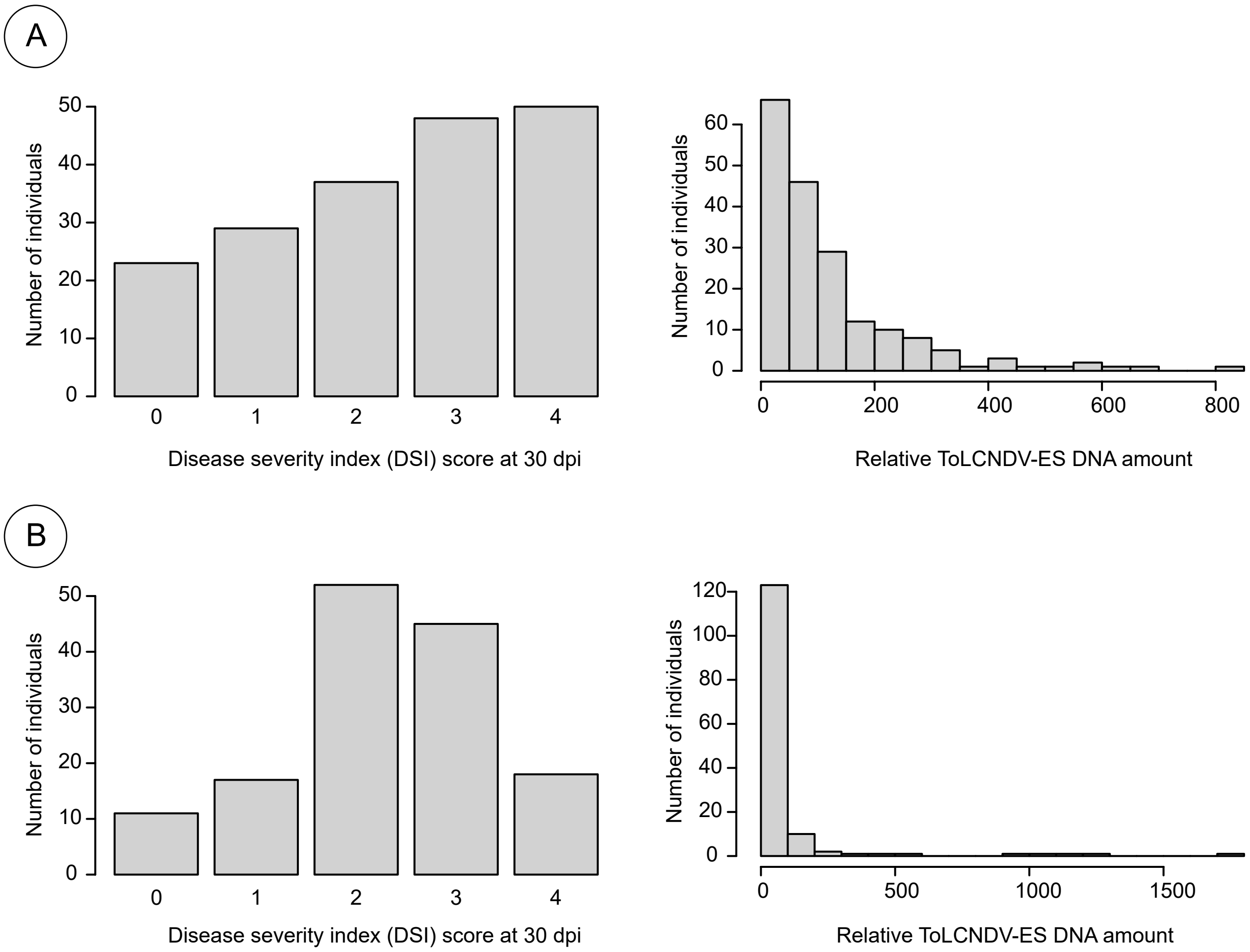

Figure S1. Phenotypic segregation of the 'Sagami Hanjiro Fushinari' (SHF) × No.44 F_2 population. Histograms of disease severity index (DSI) scores and ToLCNDV-ES viral DNA accumulation at 30 days post inoculation (dpi) (A) in the first-round SHF × No.44 F_2 population (n = 187) and (B) in the second-round SHF × No.44 F_2 population (n = 143).
