# Supplementary material for: Cy-1, a major QTL for tomato leaf curl New Delhi virus resistance, harbors a gene encoding a DFDGD-Class RNA-dependent RNA polymerase in cucumber (Cucumis sativus)
Source: BMC Plant Biol. 2024 Oct 2;24:879. doi: 10.1186/s12870-024-05591-7 (PMC11446051; doi:10.1186/s12870-024-05591-7)
Supplement: Supplementary file 2 — Supplementary Material 2. [file 12870_2024_5591_MOESM2_ESM.pdf]

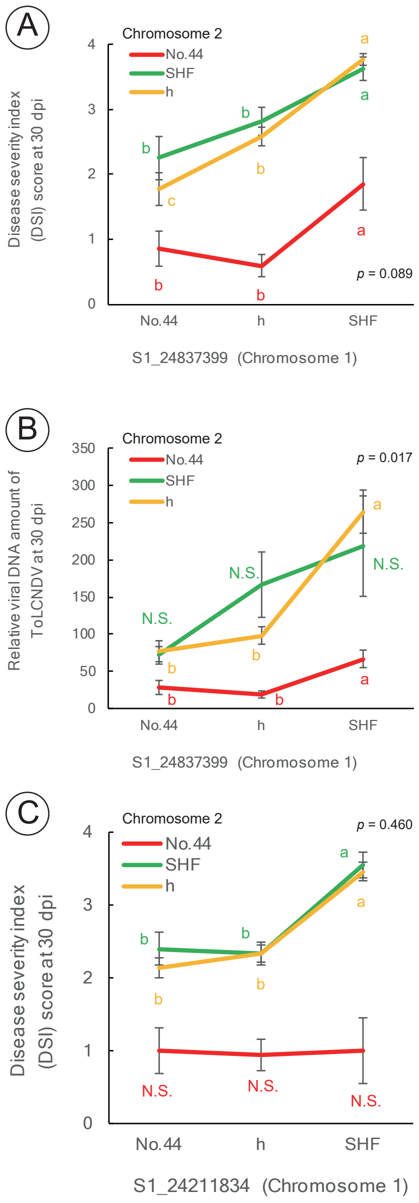

Figure S2. Effect of quantitative trait loci (QTLs) on ToLCNDV resistance in cucumber. (A) Interaction effect of chromosome 1 (S1_24837399 marker) and chromosome 2 (S2_17866822 marker) on DSI scores at 30 days post inoculation (dpi) and (B) the level of ToLCNDV-ES viral DNA accumulation at 30 dpi in the first-round 'Sagami Hanjiro Fushinari' (SHF) × No.44 F_2 population (n = 187). (C) Interaction effect of chromosome 1 (S1_24211834 marker) and chromosome 2 (S2_17695694 marker) on DSI scores at 30 dpi in the second-round SHF × No.44 F_2 population (n = 143). No.44 and SHF indicate plants homozygous for the No.44 and SHF alleles, respectively; h indicates heterozygous plants. The p-value was calculated by using a 2-way analysis of variance. Different letters within a row indicate significant differences and N.S. indicates no significance among means (ANOVA, followed by Fisher' s LSD test; α = 0.05).
