# Supplementary material for: Cy-1, a major QTL for tomato leaf curl New Delhi virus resistance, harbors a gene encoding a DFDGD-Class RNA-dependent RNA polymerase in cucumber (Cucumis sativus)
Source: BMC Plant Biol. 2024 Oct 2;24:879. doi: 10.1186/s12870-024-05591-7 (PMC11446051; doi:10.1186/s12870-024-05591-7)
Supplement: Supplementary file 3 — Supplementary Material 3. [file 12870_2024_5591_MOESM3_ESM.pdf]

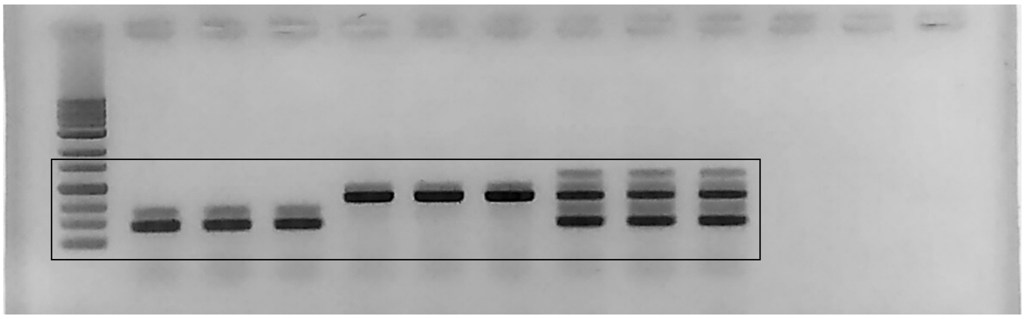

Figure S3. Original uncropped agarose gel showing the PCR amplicons of *CsRDR3* dereived from No.44, SHF, and F_1 plants using an Indel marker targeting the promoter region of *CsRDR3*. Box indicates the cropped region in Figure 5.
