# Supplementary material for: Cy-1, a major QTL for tomato leaf curl New Delhi virus resistance, harbors a gene encoding a DFDGD-Class RNA-dependent RNA polymerase in cucumber (Cucumis sativus)
Source: BMC Plant Biol. 2024 Oct 2;24:879. doi: 10.1186/s12870-024-05591-7 (PMC11446051; doi:10.1186/s12870-024-05591-7)
Supplement: Supplementary file 4 — Supplementary Material 4. [file 12870_2024_5591_MOESM4_ESM.pdf]

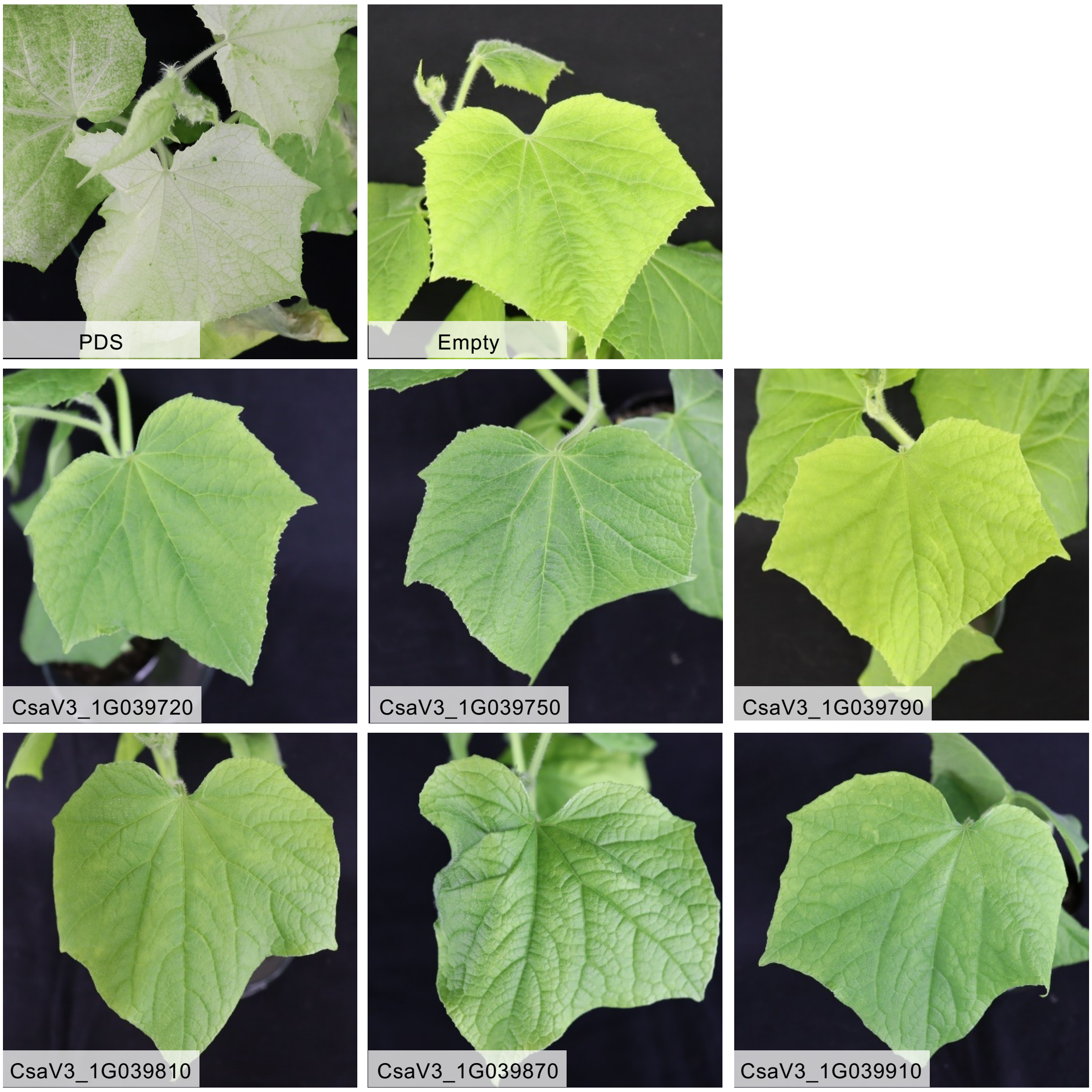

Figure S4 Functional analysis of other candidate genes by virus-induced gene silencing (VIGS). At 40 days post inoculation (dpi), no symptoms were observed in No.148 plants silenced with CsaV3_1G039720, CsaV3_1G039750, CsaV3_1G039790, CsaV3_1G039810, CsaV3_1G039870, and CsaV3_1G039910 by VIGS. Lines No.148 was homozygous for the No.44 (resistant) genotype in the target region on chromosome 1 containing *Cy-1* and for the 'Sagami Hanjiro Fushinari' (SHF; susceptible) genotype in the target region on chromosome 2 containing *cy-2*. The partial coding sequences of each gene were amplified from No.44 with primers listed in Supplementary Table S3.
