# Supplementary material for: Cy-1, a major QTL for tomato leaf curl New Delhi virus resistance, harbors a gene encoding a DFDGD-Class RNA-dependent RNA polymerase in cucumber (Cucumis sativus)
Source: BMC Plant Biol. 2024 Oct 2;24:879. doi: 10.1186/s12870-024-05591-7 (PMC11446051; doi:10.1186/s12870-024-05591-7)
Supplement: Supplementary file 5 — Supplementary Material 5. [file 12870_2024_5591_MOESM5_ESM.pdf]

Table S1 Segregation of resistant and susceptible plants in $F_1$ and $F_2$ populations derived from SHF × No.44 crosses.

| Female parent[y] | Male parent[y] | Population | Symptom segregation | | Expected ratio | $\chi^2$ test[z] |
|---|---|---|---|---|---|---|
| | | | Resistant | Susceptible | | |
| SHF (S) | No.44 (R) | $F_1$ | 0 | 31 | 0:1 | - |
| SHF (S) | No.44 (R) | $F_2$ | 52 | 135 | 1:3 | 0.7861 ($p$ = 0.38) |
| SHF (S) | No.44 (R) | $F_2$ | 28 | 115 | 1:3 | 2.2401 ($p$ = 0.13) |

[y] S, susceptible; R, resistant genotype. SHF = Sagami Hanjiro Fushinari (begomovirus susceptible cultivar).

[z] Probability of the $\chi 2$ value calculated for a recessive monogenic ratio.
