# Supplementary material for: Cy-1, a major QTL for tomato leaf curl New Delhi virus resistance, harbors a gene encoding a DFDGD-Class RNA-dependent RNA polymerase in cucumber (Cucumis sativus)
Source: BMC Plant Biol. 2024 Oct 2;24:879. doi: 10.1186/s12870-024-05591-7 (PMC11446051; doi:10.1186/s12870-024-05591-7)
Supplement: Supplementary file 7 — Supplementary Material 7. [file 12870_2024_5591_MOESM7_ESM.pdf]

Table S3 Primer list used in this study

| Primer name | Primer sequence (5'–3') | Annealing temperature | Product size and notices |
|---|---|---|---|
| Primers used for ToLCNDV viral DNA detection | | | |
| ToLCNDV A 2F | ATCTGGTGTCTCTCAACTTCC | 66.5 | For detecting ToLCNDV-[ES-Alm-Cuc-16] |
| ToLCNDV A 2R | TGGATCTAAACTTGGTGAGC | | 252 bp |
| ToLCNDV Uni F | CKWTTKCTTCTMYTTATYCKAC | 55 | For detecting ToLCNDV-[BACu-20] |
| ToLCNDV Uni R | GGCAATSGTYCCDGATAARKTC | | 353 bp |
| Primers used for ALSV viral RNA detection | | | |
| ALSV-F | CTTCTAGTTTGCATAGATCTGACCCA | 60 | For detecting ALSV. |
| ALSV-R | TTTCAAGAGTTCTCCCCCATAAGATT | | Empty: 251 bp, CsRDR3: 347 bp, CsPDS: 347 bp |
| Primers used for quantification of begomoviral DNA | | | |
| ToLCNDV A Real F | CGCATTTTCCAGACTCGTTC | 58 | For quantificating ToLCNDV-[ES-Alm-Cuc-16] |
| ToLCNDV A Real R | GCGGAACACACCACATTACA | | 113 bp |
| ToLCNDV A Real 2F | AGCTTCACGAGGATGGGTCT | 58 | For quantificating ToLCNDV-[BACu-20] |
| ToLCNDV A Real 2R | TGTTCGGATGGAAATGTGCT | | 126 bp |
| 25s-rRNA 2F | GCCCGGTCGTACTCATAACC | 58 | For quantificating the cucumber genomic DNA |
| 25s-rRNA 2R | GATCCATTTTGCCGACTTCC | | 93 bp |
| Primers used for cloning and sequencing candidate genes | | | |
| CsaV3_1G039730 full F | TGGGTTTTACAGGTATGCTGATT | 60 | For cloning and sequencing the full-length ORF |
| CsaV3_1G039730 full R | TTTACATCTTGATATACACTTATGCTT | | of *CsRDR3* |
| CsRDR CO1 | CTCATCTGCAAAGGGTTTTAGG | 60 | |
| CsRDR CO2 | ATAGCTCGTTCTCCAACTCCTC | 60 | |
| CsRDR CO3 | AGCTGATGTGGTTGGATTCTTC | 60 | |
| Primers used for qPCR analysis of *CaRDR3a* | | | |
| CsRDR real 2F | AAAAGAGCTTGAAAAGCGGAAG | 58 | For analyzing *CsRDR3* expression |
| CsRDR real 2R | CCCGTTGATTTGTCCATTCTC | | 128 bp |
| CsActin2 Real F | GGCATTGCAGACAGGATGAG | 58 | For analyzing *CsActin* expression |
| CsActin2 Real R | TGTATTTCCTTTCGGGTGGTG | | 91 bp |
| Primers use for constracting VIGS vector | | | |
| CsRDR-189-Xho | TACATCTCGAGTCAACTTGCTCTGTTTCTTTGC | 60 | For cloning *CsRDR3* (CsaV3_1G039730) to ALSV |
| CsRDR-189-Bam | TACATGGATCCTCTAAACTCAAGTTCTCCCAAAGC | | 189 bp |
| CsRDR3-102-Xho | TACATCTCGAGGTTCTTGTTTATCGCAATCCT | 60 | For cloning *CsRDR3* (CsaV3_1G039730) to ALSV |
| CsRDR3-102-Bam | TACATGGATCCATATTTTGCATTTCCAACGACA | | 102 bp |
| CsPDS_ver.2_Xho | TACATCTCGAGAATCCTCCTGAGAGACTATGTG | 60 | For cloning *CsPDS* to ALSV |
| CsPDS_ver.2_ Bam | TACATGGATCCATTGTTCAACTCAATTTTTTGTATC | | 102 bp |
| CsaV3_1G039720 VIGS F | TACATCTCGAGTTTGGTTGTCATCAAGAAGCTG | 60 | For cloning CsaV3_1G039720 to ALSV |
| CsaV3_1G039720 VIGS 2R | TACATGGATCCAGGACCTCCATCCCCACACT | | 102 bp |
| CsaV3_1G039750 VIGS F | TACATCTCGAGTCTCAGGTGGCCAAATTCTGT | 60 | For cloning CsaV3_1G039750 to ALSV |
| CsaV3_1G039750 VIGS 2R | TACATGGATCCTTCCCATCTCTTCTGCCATTC | | 102 bp |
| CsaV3_1G039790 VIGS F | TACATCTCGAGTTGTATGTGCAAGAAGGGATTG | 60 | For cloning CsaV3_1G039790 to ALSV |
| CsaV3_1G039790 VIGS 2R | TACATGGATCCTAATGCATTCTTTTCAGCCAACTTA | | 102 bp |
| CsaV3_1G039810 VIGS F | TACATCTCGAGATGGTCTACCTCCGTCAGAAGG | 60 | For cloning CsaV3_1G039810 to ALSV |
| CsaV3_1G039810 VIGS 2R | TACATGGATCCGGCGGAAGCGCCGAC | | 102 bp |
| CsaV3_1G039910 VIGS F | TACATCTCGAGCAGGGTGCTCGTGTAATCTTTA | 60 | For cloning CsaV3_1G039910 to ALSV |
| CsaV3_1G039910 VIGS 2R | TACATGGATCCTATGGCTGCAAGATCAGTTGAA | | 102 bp |

K = G or T; M = A or C; R = A or G; S = C or G; W = A or T; Y = C or T; D = A, G, or T
