# Supplementary material for: Cy-1, a major QTL for tomato leaf curl New Delhi virus resistance, harbors a gene encoding a DFDGD-Class RNA-dependent RNA polymerase in cucumber (Cucumis sativus)
Source: BMC Plant Biol. 2024 Oct 2;24:879. doi: 10.1186/s12870-024-05591-7 (PMC11446051; doi:10.1186/s12870-024-05591-7)
Supplement: Supplementary file 8 — Supplementary Material 8. [file 12870_2024_5591_MOESM8_ESM.pdf]

Table S4 Conditions for PCR in this study

	Temperature	Time	
ToLCNDV-ES (ToLCNDV-[ES-Alm-Cuc-16])viral DNA detection			
	94 °C	2 min	
	98 °C	10 sec	35 cycles
	66.5 °C	30 sec	
	72 °C	1 min	
	72 °C	3 min	
ToLCNDV-IDN (ToLCNDV-[BACu-20]) viral DNA detection			
	94 °C	2 min	
	98 °C	10 sec	35 cycles
	55 °C	30 sec	
	72 °C	1 min	
	72 °C	3 min	
Quantification of ToLCNDV titer by real-time PCR			
	95 °C	2 min	
	95 °C	5 sec	40 cycles
	58 °C	10 sec	
Fine mapping			
SNP analysis by HRM			
	95 °C	2 min	
	95 °C	15 sec	40 cycles
	55.6 °C	20 sec	
	60 °C → 95 °C	(increment of 0.2 °C)	
Indel analysis			
	94 °C	2 min	
	94 °C	10 sec	35 cycles
	60 °C	30 sec	
	72 °C	1 min	
	72 °C	3 min	
Expression analysis of *CsRDR3* and *CsActin* by qPCR			
	95 °C	2 min	
	95 °C	5 sec	40 cycles
	58 °C	10 sec	
