# Supplementary material for: Cy-1, a major QTL for tomato leaf curl New Delhi virus resistance, harbors a gene encoding a DFDGD-Class RNA-dependent RNA polymerase in cucumber (Cucumis sativus)
Source: BMC Plant Biol. 2024 Oct 2;24:879. doi: 10.1186/s12870-024-05591-7 (PMC11446051; doi:10.1186/s12870-024-05591-7)
Supplement: Supplementary file 9 — Supplementary Material 9. [file 12870_2024_5591_MOESM9_ESM.pdf]

Table S5 DNA markers developed in this study

Primer name	Primer sequence (5'–3')	Annealing temperature	SNP or indel position in reference sequence of Zunla	Mutation	Product size
KASPar assay					
Cuc24327273-KASP C1	TGAAATTCGTGATGCCTA		Chr01: 24327273	No.44: G	63 bp
Cuc24327273-KASP A1	GAAGGTGACCAAGTTCATGCTAACGGAGCAGACGATAA			SHF: A	63 bp
Cuc24327273-KASP A2	GAAGGTCGGAGTCAACGGATTGAACGGAGCAGACGATAG				
Cuc24421202-KASP C1	GTTGGGATCAGGTTAGTCA		Chr01: 24421202	No.44: G	62 bp
Cuc24421202-KASP A1	GAAGGTGACCAAGTTCATGCTTTTCATAACCACAATAAGCTATATTAA			SHF: A	62 bp
Cuc24421202-KASP A2	GAAGGTCGGAGTCAACGGATTTTTCATAACCACAATAAGCTATATTAG				
Cuc24511076-KASP C1	CTAAAGTCAATCTCAAACTAATAACAG		Chr01: 24511076	No.44: G	60 bp
Cuc24511076-KASP A1	GAAGGTGACCAAGTTCATGCTACGTCTGAATGTTGATTCAT			SHF: T	60 bp
Cuc24511076-KASP A2	GAAGGTCGGAGTCAACGGATTACGTCTGAATGTTGATTCAG				
Cuc24714468-KASP C1	TGTCATGTAAGGCCTAACC		Chr01: 24714468	No.44: A	55 bp
Cuc24714468-KASP A1	GAAGGTGACCAAGTTCATGCTTTTGGTTTAGGGATATGTGATA			SHF: G	55 bp
Cuc24714468-KASP A2	GAAGGTCGGAGTCAACGGATTTGGTTTAGGGATATGTGATG				
Cuc24805665-KASP C1	CTATTATACCTTCTCAAGCAACC		Chr01: 24805665	No.44: G	65 bp
Cuc24805665-KASP A1	GAAGGTGACCAAGTTCATGCTTACGAAACTACAAATTTACCTGA			SHF: A	65 bp
Cuc24805665-KASP A2	GAAGGTCGGAGTCAACGGATTCGAAACTACAAATTTACCTGG				
Cuc24895001-KASP C1	AAGATATGCCATGAGTATCATAAG		Chr01: 24895001	No.44: C	61 bp
Cuc24895001-KASP A1	GAAGGTGACCAAGTTCATGCTTGATAAAATTCAGGCACTCA			SHF: T	61 bp
Cuc24895001-KASP A2	GAAGGTCGGAGTCAACGGATTGATAAAATTCAGGCACTCG				
Cuc24978218-KASP C1	CAGGTAAGTAATTTGCTTACCA		Chr01: 24978218	No.44: T	61 bp
Cuc24978218-KASP A1	GAAGGTGACCAAGTTCATGCTATAGGAGGGATAGGGTGAC			SHF: C	61 bp
Cuc24978218-KASP A2	GAAGGTCGGAGTCAACGGATTGATAGGAGGGATAGGGTGAT				
Cuc25193180-KASP C1	ATCTCAATCTCGGTGCA		Chr01: 25193180	No.44: A	60 bp
Cuc25193180-KASP A1	GAAGGTGACCAAGTTCATGCTGACAACCGAGAAATCGAT			SHF: C	60 bp
Cuc25193180-KASP A2	GAAGGTCGGAGTCAACGGATTGACAACCGAGAAATCGAG				
Cuc25270268-KASP C1	CAAGACAATTTGGGAACATA		Chr01: 25270268	No.44: T	52 bp
Cuc25270268-KASP A1	GAAGGTGACCAAGTTCATGCTGTCCAAATATCCAGAATCGT			SHF: C	52 bp
Cuc25270268-KASP A2	GAAGGTCGGAGTCAACGGATTCCAAATATCCAGAATCGC				
Cuc25380586-KASP C1	GAGCACCAAAGCCATC		Chr01: 25380586	No.44: C	57 bp
Cuc25380586-KASP A1	GAAGGTGACCAAGTTCATGCTTTTCAATCATTGGGATTGT			SHF: T	57 bp
Cuc25380586-KASP A2	GAAGGTCGGAGTCAACGGATTTCAATCATTGGGATTGC				
SNP HRM analysis					
Cuc25061147-HRM F	TGGCAATAAACAAATCAAAAC	55.6	Chr01: 25061147	No.44: G	140 bp
Cuc25061147-HRM R	CCTCCCATTGTACAAAGTTC			SHF: A	140 bp
Cuc25133958-HRM F	CTGACATGGAGTTTTCCAAT	55.6	Chr01: 25133958	No.44: G	154 bp
Cuc25133958-HRM R	CTGCAGAATCTTAGCCCTTA			SHF: A	154 bp
Cuc25183282-HRM F	TGAAAGCAAAGCTATTACCAG	55.6	Chr01: 25183282	No.44: A	148 bp
Cuc25183282-HRM R	TAGAATCTGAACGCTGTTTG			SHF: C	148 bp
Cuc25189963-HRM F	GCCTTCCATTTTCTCAAAG	55.6	Chr01: 25189963	No.44: T	142 bp
Cuc25189963-HRM R	AGCAATCAAAAGTGGAGAAC			SHF: G	142 bp
Cuc25238586-HRM F	CCAAATTACATGGCGAATAC	55.6	Chr01: 25238586	No.44: A	140 bp
Cuc25238586-HRM R	ACCCCACTAAGACTGTTCCT			SHF: G	140 bp
Indel analysis					
RDR upstream F	TATATTAATCAATTTCCCTTTTGC	60	Chr01: 25119503	No.44	454 bp
RDR upstream 2F	AATGTAAATTTGTTGCATTCTCTT			SHF	805 bp
RDR upstream R	GAAAAACAGTTCCTTTTATCCTC			1924 bp deletion and 62 bp insertion in No.44	
